# Neutrophil extracellular traps mediate the crosstalk between plaque microenvironment and unstable carotid plaque formation

**DOI:** 10.1038/s12276-024-01281-4

**Published:** 2024-08-01

**Authors:** Yu Cao, Minghui Chen, Xinyu Jiao, Shuijie Li, Dong Wang, Yongxuan Zhan, Jiaju Li, Zhongfei Hao, Qingbin Li, Yang Liu, Yan Feng, Ruiyan Li, Hongjun Wang, Mingli Liu, Qiang Fu, Yongli Li

**Affiliations:** 1 Department of Neurosurgery, The Second Affiliated Hospital of Harbin Medical University, Harbin, 150086 China; 2 Department of Ultrasound, The Second Affiliated Hospital of Heilongjiang University of Chinese Medicine, Harbin, 150006 China; 3 Department of Biopharmaceutical Sciences, College of Pharmacy, Harbin Medical University, Harbin, 150076 China; 4 State Key Laboratory of Frigid Zone Cardiovascular Diseases (SKLFZCD), Harbin, China; 5 Scientific Research Centre, The Second Affiliated Hospital of Harbin Medical University, Harbin, 150086 China; 6 Department of Chinese Formulae, Heilongjiang University of Chinese Medicine, Harbin, 150040 China

**Keywords:** Computational biology and bioinformatics, Cell biology, Immunology, Molecular biology

## Abstract

The development of unstable carotid atherosclerotic plaques is associated with the induction of neutrophil extracellular traps (NETs) via the activation of diverse inflammatory mediators in the circulating bloodstream. However, the underlying mechanisms through which NETs influence the microenvironment of atherosclerotic plaques and contribute to the development of unstable carotid plaques remain largely elusive. The objective of this study was to elucidate the role of myeloid differentiation protein 1 (MD-1, *LY86*)-induced NETs underlying the crosstalk between unstable plaque formation and the plaque microenvironment. We employed bioinformatics analysis to identify key genes associated with carotid-unstable plaque, followed by comprehensive validation using various experimental approaches on tissue specimens and plasma samples classified based on pathological characteristics. Patients with carotid-unstable plaques exhibited elevated plasma concentrations of MD-1 (*LY86*), while patients with stable plaques demonstrated comparatively lower levels. Furthermore, soluble MD-1 was found to induce the formation of NETs through activation of Toll-like receptor signaling pathway. The proliferative and immature vascularization effects of NETs on endothelial cells, as well as their inhibitory impact on cell migration, are directly correlated with the concentration of NETs. Additionally, NETs were found to activate the NF-κB signaling pathway, thereby upregulating ICAM1, VCAM1, MMP14, VEGFA, and IL6 expression in both Human umbilical vein endothelial cells (HUVECs) and HAECs. Subsequently, a significant increase in intraplaque neovascularization by NETs results in poor carotid plaque stability, and NETs in turn stimulate macrophages to produce more MD-1, generating a harmful positive feedback loop. Our findings suggest that soluble MD-1 in the bloodstream triggers the production of NETs through activation of the Toll-like receptor signaling pathway and further indicate NETs mediate a crosstalk between the microenvironment of the carotid plaque and the neovascularization of the intraplaque region. Inhibiting NETs formation or MD-1 secretion may represent a promising strategy to effectively suppress the development of unstable carotid plaques.

## NETs influence carotid plaque microenvironment and neovascularization

Atherosclerosis, a disease where arteries get blocked with fat, is a main cause of heart disease and stroke. Predicting which atherosclerotic plaques will cause heart attacks is hard. Researchers analyzed gene data from unstable and stable carotid plaques, focusing on neutrophils and a protein called MD-1. The study involved 30 patients and 10 healthy volunteers to understand how MD-1 and neutrophils contribute to plaque instability. The main finding is that MD-1 could be a biomarker for unstable plaques, offering a new target for therapies to prevent major heart events. This progress in understanding the molecular mechanisms behind plaque instability could lead to better prevention strategies for heart disease. Future research may focus on developing treatments that target MD-1 and neutrophils to stabilize plaques and reduce the risk of heart attacks. This summary was initially drafted using artificial intelligence, then revised and fact-checked by the author.

## Introduction

As the global population ages, cardiovascular and cerebrovascular disease has become a major public health concern^1^. Atherosclerosis (AS) is an immune-mediated disease that is a major underlying cause of cardiovascular disease^2^. Unstable plaque is an evolving concept of an elevated risk atherosclerotic plaque with a risk of rapid stenosis progression and a propensity for thrombosis, and there is no definitive evidence to prospectively identify unstable plaques^3^. Therefore, the assessment of atherosclerotic plaque instability and early intervention are essential for plaque risk stratification and the reduction of major adverse cardiovascular and cerebrovascular events^4^.

Carotid atherosclerotic plaque formation involves complex immune and inflammatory responses. The microenvironment of atherosclerotic plaques is a highly complex physical and biochemical environment involving several cell types and molecules, such as immune cells, endothelial cells (ECs), inflammatory factors, and chemokines^5^. Neutrophils are the most abundant immune cells involved in the development of carotid plaques. In addition to phagocytosis, neutrophils play an essential role in defense against pathogenic microorganisms through the release of neutrophil extracellular traps (NETs)^6^. NETs are extracellular reticulated fibrous structures that are excreted from activated neutrophils in response to a variety of inflammatory stimuli and are composed of histone proteins, DNA, nuclear chromatin, and granule proteins (e.g., elastase, historian, and myeloperoxidase)^7^. NETs are cytotoxic and thrombogenic and may trigger the formation of atherosclerotic plaques and arterial thrombosis^8^. Neutrophils express most members of the TLR family, including TLR4. The activation of cell surface TLRs has multiple effects on the functions of neutrophils, including cytokine production, reactive oxygen species (ROS) production, receptor expression, and phagocytosis^9^.

Myeloid differentiation protein 1 (MD-1), also known as lymphocyte antigen 86 (*LY86*), is predominantly expressed in immune cells, including macrophages, B cells, and dendritic cells^10^. MD-1 is a key regulator of heart failure, ischemia-reperfusion, chronic inflammation in adipose tissue, obesity, and insulin resistance, and MD-1 may be a candidate modifier gene for a variety of inflammatory and atopic diseases^11^. Soluble MD-1 is a valuable biomarker for inflammatory diseases, and soluble MD-1 can bind to the TLR4 receptor^12^. Therefore, we propose that soluble MD-1 may interact with the TLR4 receptor on neutrophils, which subsequently activates downstream signaling pathways and induces the formation of NETs. The microenvironment of unstable plaques is weakly acidic (pH 6.0-6.8) and enriched with ROS, lipids, and enzymes^13^. In addition, pathologic intimal thickening and inflammatory cell infiltration result in a hypoxic environment and stimulate neovascularization as a compensatory mechanism^14^. Neovascularization predisposes to plaque hemorrhage and rupture^15^. NETs were found to play a role in proinflammatory activation and proangiogenic responses in vitro and in vivo in previous studies^16^. However, the impact of NETs on intraplaque neovascularization and the related mechanism remains incompletely understood. Therefore, we propose for the first time that NETs may disrupt plaques by modulating the plaque microenvironment and facilitating enhanced neovascularization within the plaque. Through a combination of bioinformatics analyses and experimental investigations, we elucidated that MD-1 (*LY86*) has potential as a predictive marker for unstable plaques and represents an ideal target for inhibiting NETs production and preventing unstable plaque formation.

## Materials and methods

### Bioinformatics analysis

In this study, we downloaded the raw gene expression data from the GSE28829, GSE41571, GSE43292, GSE179828, GSE145200, and GSE3037 datasets obtained from the GEO database (https://www.ncbi.nlm.nih.gov/geo/). We merged and preprocessed the raw data using the sva package in R software (R-project.org), which included background adjustment, normalization, and logarithmic transformation, using a robust multiarray averaging method. The synthetic datasets were normalized using “limma” and “pheatmap” in R. Differentially expressed genes were screened and clustered with *P* < 0.05 and |fold change| ≥ 1. Differentially expressed genes in the GSE28829 dataset were screened and clustered. Late plaque samples in GSE28829, ruptured plaque samples in GSE41571, and unstable plaque samples in GSE43292 were classified into the unstable plaque group, while early plaque samples, stable plaque samples, and macroscopically intact tissues in each of the above datasets were classified into the stable plaque group. Samples from the GSE179828 dataset were classified into the stimulus group of NETs and the control group of HUVECs and NTs. The NETs samples in the GSE145200 dataset were classified as NETs pos in the positive group and NETs neg in the negative group. The GSE3037 dataset was derived from human peripheral blood neutrophils. Control individuals (Patient control) were categorized as the control group, and HMGB1-treated patients (Patient HMGB1 treated) and LPS-treated patients (Patient LPS treated) were categorized as the experimental group. Gene Ontology (GO) enrichment analysis of enrichment of GO terms, including biological process (BP), cellular component (CC), and molecular function (MF) terms, and KEGG pathway enrichment analysis were performed using the cluster analysis package in R. A p.adjust value < 0.05 and *q* value < 0.2 were considered to indicate significant differences. An xCell score based on the xCell analysis method in R was used to assess the enrichment of each cell type. Weighted gene co-expression network analysis (WGCNA) was performed using the WGCNA package in R to identify co-expression networks and cluster genes into different modules. Genes in the top 75% of the normalized variance were selected from the dataset for the next experiment. We selected the soft threshold power (β) based on the scale-free topology criterion using the selection soft threshold function. Then, a multistep network construction method was used to identify gene co-expression modules. Each module had at least 100 genes. A threshold of 0.6 was set for the merged modules to plot the inter-module correlations using the dynamic cut-tree method. Data on immune-infiltrating cells that differed significantly among all samples according to the xCell calculation were combined. The samples are clustered, and the correlation between the model feature matrix and the sample information matrix was calculated. The genes associated with neutrophils were identified and downloaded from the GeneCards (https://www.genecards.org/) website. After the intersection of deg&purple&neutrophils, 57 genes were imported into the STING database to construct a PPI network with a minimum interaction score of 0.4. Cytoscape (v3.9.1) was then utilized to construct a visual PPI network and identify hub genes. The top 10 genes in GSE43292 were screened to validate and evaluate the diagnostic and discriminative values of the genes using subject work characteristic (ROC) curve analysis. Finally, the GSEA method was used to identify the enriched marker gene set pathways (*p*-value < 0.05, FDR < 0.05) between the screened unstable plaques and stable plaques, and the marker gene sets used were obtained from the Molecular Signatures Database (MSigDB).

### Patient characteristics and sample collection

A total of 30 patients with carotid artery stenosis due to carotid atherosclerotic plaque and 10 normal volunteers were enrolled at the Second Affiliated Hospital of Harbin Medical University from August 2020 to November 2022. Fresh venous blood samples from patients on the first day of hospitalization and venous blood from healthy volunteers were collected, and plasma was collected after centrifugation and frozen at -80 °C. Twenty-nine of the patients were treated with carotid endarterectomy (CEA). Atherosclerotic plaques were obtained during the procedure, and the intact plaques were cut into two sections. One section was preserved in 4% paraformaldehyde for pathological staining, immunohistochemistry and immunofluorescence, and the remaining section was frozen in liquid nitrogen for RNA and protein extraction.

### Histopathologic examination and classification of carotid plaques in patients

Hematoxylin (H8070, Solarbio, China), eosin (A600190, Sangon, China), oil red O (O0625, Sigma, USA), and Masson trichrome (p8330, 71019360, Sinopharm, China) were used to stain the tissue sections. Carotid plaque staining results were observed and photographed using an Olympus BX53 microscope. Plaques were classified as stable or unstable according to clinical and histologic criteria^17^ (Supplementary Table 1). Classification of the carotid plaque was performed by two independent investigators. The validation experiment was approved by the local ethics committee (YJSKY2022-149), and consent was obtained from all patients to participate in the study.

### Validation with reverse transcription-quantitative polymerase chain reaction (RT‒qPCR)

A fluorescence quantitative PCR instrument (Exicycler 96, BIONEER, Korea) was used for the validation of four candidate hub genes (LY86, ITGB2, CCR1, and CSF1R) by RT‒qPCR. Total RNA was extracted from 10–50 mg of plaques using TRIpure (RP1001, BioTeke, Beijing, China) and reverse transcribed into complementary DNA (cDNA) using BeyoRT II M-MLV reverse transcriptase (D7160L, Biyuntian, Shanghai) according to the instructions. cDNA was amplified using SYBR Green 2x Taq PCR MasterMix (PC1150, Solarbio, Beijing, China) and 0.4 μmol of each primer pair (Anhui General Bio Co., Ltd., China). β-actin was used as an internal control, and the expression of the candidate genes was calculated by the 2−ΔΔCt method. The sequences of the primers used for the RT‒qPCR analysis are shown in Supplementary Table 2.

### Immunohistochemistry

Immunohistochemistry was used to assess the expression of MD-1 (*LY86*). Primary antibodies against MD-1 (1:50, A6185, ABclonal, China) were incubated overnight at 4 °C. Sections were incubated with horseradish peroxidase-labeled secondary antibodies and then stained with DAB. Images were taken at 40× to calculate the MD-1-positive areas in the plaques. The integrated optical density (IOD) of the target genes and the total tissue area were measured using Image-Pro Plus 6.0 software (IPP 6.0, Media Control Sciences, USA). The gene expression intensity was expressed as the IOD per unit area.

### ELISA

The plasma MD-1 levels of 30 patients and 10 healthy volunteers were determined using a commercial enzyme-linked immunosorbent assay (ELISA) MD-1 Kit (ZC-55707, Shanghai Thrive Biotechnology Co., Ltd., China) according to the manufacturer’s instructions, with three replicate wells for each sample. A human NETs kit (mm-2479H1) was used to determine the level of NETs produced by neutrophils induced in vitro, and a murine NETs kit (MM-1183M2), murine MD-1 kit (MM-47021M2), murine ICAM1 kit (MM-0183M2), murine VCAM1 kit (MM-0129M2), murine MMP14 kit (MM-46773M2), mouse VEGFA kit (MM-44452M2) and mouse IL6 kit (MM-0163M2, Jiangsu Enzyme Immunity Industry Co., Ltd., China) were used to determine the expression levels of the proteins in mouse plasma.

### Isolation of human peripheral blood neutrophils

After providing informed consent, fresh blood was drawn from healthy volunteers (males and females, 20–30 years old) by venipuncture. The research protocol was approved by the ethics committee of Harbin Medical University. Neutrophils were isolated using a human peripheral blood neutrophil isolation solution kit (P9040, Solarbio, Beijing, China) and then resuspended in RPMI 1640 medium supplemented with or without 2% fetal bovine serum (FBS, SA101, CellMax, Lanzhou, China). The neutrophil viability was >95%, as assessed by the Trypan blue dye exclusion test, and the neutrophil purity was >95%, as assessed by Giemsa staining.

### Quantification of NETs releases

Fresh human neutrophils (1 × 10^5^ cells per well) were inoculated into 6-well plates and 6-well plates precoated with 0.001% polylysine L and incubated with different concentrations of recombinant human MD-1 (novoprotein, Suzhou, China) in the presence of 2% FBS. The concentrations of MD-1 were determined according to the results of the previous assays as 25, 50, 75, 100, and 125 ng/ml, and the specified continuous induction times were 1, 2, 4, 6, 8, 10, and 12 h at 37 °C and 5% CO_2_. A 1:1 mixture of deionized water and dimethyl sulfoxide (DMSO) was used as a blank control, and phorbol myristate^18^ (PMA, 30 nM, P3189, Sigma, USA) and lipopolysaccharide^19^ (LPS, 30 ng/mL, Sigma, USA) were used as positive controls. After induction, the solution in each well was mixed by gentle blowing and collected by centrifugation (200 × *g*, 5 min), and the supernatant was collected to determine the NETs concentration by ELISA. After reaching the appropriate induction concentration, the cells were pretreated with selected inhibitors (1 μM TAK-242, 10 μM ST2825, 0.5 μM IRAK1-4 INH, 10 μM C25-140, 10 μM SB203580, 10 μM U0126, and 10 μM DPI; Apexbio, USA) for 1–4 h before being assayed again for the determination of the NETs concentration.

### Hoechst staining

After the above treatment, liquid was aspirated from the coated 6-well plates, the plates were stained with a Hoechst 33342 Staining Kit (C0003, Beyotime, China), and the structure of the NETs in five random fields of view was observed and photographed using a fluorescence microscope.

### Immunofluorescence

Samples from the neutrophil inhibitor assay group, clinical plaque tissue, and animal tissue described above were processed, fixed in 4% paraformaldehyde for 15 min, and permeabilized with Triton X-100 (A2576, ALPHABIO, Tianjin, China) for 30 min at room temperature. Nonspecifically bound sites were blocked with 50% goat serum in PBS at 37 °C for 30 min. Neutrophil samples were incubated overnight at 4 °C with a murine anti-myeloperoxidase antibody (1:100, ab90810, Abcam, USA), and tissue and animal samples were incubated with an anti-CD31 antibody (1:50, 66065-2-Ig, Proteintech, Wuhan, China), followed by incubation with an Alexa Fluor 555-conjugated secondary antibody (1:500, Beyotime, Shanghai, China). DNA was labeled with an anti-fluorescence burst sealer containing DAPI (50 μL, AL739A, ALPHABIO, Tianjin, China). Images were acquired using fluorescence microscopy. Tissue and animal samples were observed and photographed under a confocal microscope, and the microvessel density (MVD) was calculated. The calculation of the MVD required three randomly selected high-magnification (20×) fields of view from three different sections of the tissue samples, and CD31-positive luminal or nonluminal ECs in the field of view were observed and counted. The microvessel density, expressed as the average number of microvessels per field of view, was quantified by two individuals who were unaware of the experimental design.

### Co-immunoprecipitation

To determine the interaction between MD-1 and TLR4, we performed co-immunoprecipitation (Co-IP). Temporally, neutrophils were incubated with recombinant human MD-1 (75 ng/mL) for 60 min at 37 °C in 5% CO_2_. After incubation, the cells were washed twice with PBS, and total cellular proteins were extracted using Western and IP Cell Lysis Buffer (P0013, Beyotime, Shanghai, China) containing protease inhibitors, followed by centrifugation at 14,000 × *g* for 5 min at 4 °C. Experiments were performed using an immunoprecipitation kit (Protein A magnetic bead assay, P2175S, Beyotime, Shanghai, China) according to the instructions. Briefly, 50 μg/ml of mouse anti-MD-1 antibody (SANTA CRUZ BIOTECHNOLOGY, INC., USA) and 50 μg/ml of mouse anti-TLR4 antibody (Proteintech, Wuhan, China) were conjugated to Protein A magnetic beads and separated from the magnetic beads. Then, the samples were incubated with Protein A magnetic beads conjugated with antibodies or normal IgG, and the samples were magnetically separated and washed after SDS‒PAGE. Proteins transferred to PVDF membranes were imputed using mouse anti-MD-1 antibody and mouse anti-TLR4 antibody and then incubated with HRP-coupled secondary antibody (1:5000, ZSGB-BIO, Beijing, China). The presence of MD-1 and TLR4 in the immunoprecipitates was detected by HRP-coupled goat anti-mouse IgG secondary antibody (1:5000, AS003, ABclonal, Wuhan, China).

### Duolink proximity ligation assay

To detect the interaction between MD-1 and TLR4, we used the Duolink^®^ In Situ Proximity Ligation Assay Kit (DUO92101, Sigma, USA). Freshly isolated neutrophils from carotid plaque patients and healthy volunteers were inoculated in 24-well plates, in which healthy volunteer neutrophils were incubated with recombinant human MD-1 (50 ng/ml and 75 ng/mL) and MD-1 (75 ng/ml)&TAK-242 (1 μM) for 60 min at 37 °C with 5% CO_2_. After treatment, neutrophils from each group were fixed with 4% paraformaldehyde for 15 min at room temperature, treated with 0.5% Triton X-100 for 30 min, and blocked with 1x blocking solution for 60 min at 37 °C. The cells were then incubated with two primary antibodies against MD-1 and TLR4 for 3 h at 37 °C. After washing, the cells were incubated with the mixed PLA probe for 60 min at 37 °C. This pair of secondary antibodies produces a signal only when the two probes are in close proximity (<40 nm). After washing again, the cells were ligated, amplified, and finally assayed separately. Fluorescence images were acquired under a fluorescence microscope.

### Cell culture

HUVECs (ATCC, Manassas, USA) and HAECs (HTX23972, Haodi Huatuo Biotechnology Co., Ltd., Shenzhen, China) were incubated at 37 °C under 5% CO_2_ in DMEM-F12 or endothelial cell medium (HTX23972, Zhong Qiao Xin Zhou Biotechnology Co., Ltd., Shanghai, China) supplemented with 10% FBS and 100 nmol/L penicillin/streptomycin (C0222, Beyotime, Shanghai, China) for 5 days. All assays were performed using cells from generation 6 and below.

### Generation, isolation, and preparation of NETs and NETs culture media

Purified neutrophils (1 × 10^6^/well) were seeded in 6-well plates, stimulated with 50 ng/ml MD-1 or 75 ng/ml MD-1, and incubated at 37 °C and 5% CO_2_ for 4 h. The medium was then gently removed, leaving the NETs and neutrophils attached to the plate. Precooled PBS without calcium and magnesium was added to the eluted NETs and neutrophils. The liquid in the 6-well plates was collected and centrifuged at 450 × *g* at 4 °C for 10 min, after which the supernatant was collected. The supernatant was collected again by centrifugation at 15,000 × *g* for 15 min at 4 °C and its concentration was determined. Preparations were cultured in DMEMF12 medium containing 2% FBS containing low and elevated concentrations of NETs (as described above), NETs, and DNase I (100 U/mL, 10104159001, Sigma, USA) or ddH_2_O and DMSO. Cultures were incubated at 37 °C and 5% CO_2_ for 48 h, after which DNase I was used to pretreat the NETs for 1 h at 37 °C before subsequent assays.

### Protein extraction and Western blot analysis

Neutrophils (5 × 10^6^/ml) were treated with recombinant human MD-1 (75 ng/mL) and maintained at 37 °C with 5% CO_2_ for 15 min, 30 min, 45 min, or 1 h. The cells were also induced by the addition of MD-1 (75 ng/mL) for 3 h after the grouping of each of the previously mentioned inhibitors. HUVECs and HAECs were treated with medium containing NETs, NETs, and BAY11-7082 (5 μM, HY-13453, MedChemExpress, Shanghai, China) or ddH_2_O, DMSO, and BAY11-7082. Carotid plaque tissues, neutrophils, HUVECs, and HAECs were washed twice with cold PBS and lysed with RIPA buffer (P0013B, BAY11, Shanghai, China) containing protease inhibitor (K1007, Apexbio, USA) and phosphatase inhibitor (K1015, Apexbio, USA). The total protein concentration was determined using a BCA Protein Assay Kit (P0010S, Biyuntian, Shanghai). Equal amounts of the proteins were separated by SDS‒PAGE and transferred to a PVDF membrane. After blocking (P0252, Biyun Tian, Shanghai, China), each sample membrane was incubated with primary antibodies against various candidate proteins, such as MD-1 (1:100, sc-390613, Santa Cruz Biotechnology, USA), CCR1 (1:2000, abs105357, Absin, Shanghai, China), p-IRAK1 (1:500, bs3192R), p-IRAK4 (1:500, bs4080R, Beijing Boaoxian Biotechnology Co. ab180747), IRAK4 (1:1000, ab32511), p38 MAPK (1:1000, ab170099), p-p38 MAPK (1:1000, ab195049), ERK1/2 (1:10000, ab184699), p-ERK1/2 (1:10000 ab278538, Abcam, USA), CSF1R (1:500, A3019), ITGB2 (1:500, A19012), ICAM1 (1:2000, A22596), VCAM1 (1:100, A0279), MMP14 (1:500, A0067), VEGFA (1:500, A5708), IL6 (1:500, A22222), TLR4 (1:500, A0007), NF-κB (1:2000, A22331), p-NF-κB (1:500, AP0838), and β-actin (1:100000, AC026, ABclonal, Wuhan, China) were incubated at 4 °C overnight. Then, a specific HRP-conjugated secondary antibody (1:10000, ZB-5301, Zhongshi Jinqiao, Beijing, China) was used. Immunoreactive bands were detected with an enhanced chemiluminescence (ECL) substrate (BL520A, Biosharp, Hefei, China). Quantitative blotting was performed with ImageJ.

### Cell proliferation and colony formation assay

A cell counting kit-8 (CCK-8, K1018, Apexbio, USA) was used to determine the relative cell growth at different time intervals (4, 8, 12, 24, and 48 h). The absorbance (optical density at 450 nm) of each 96-well plate was measured using an enzyme marker. In the colony formation assay, HUVECs resuspended in the various media mixtures described above were seeded in 6-well plates (200 cells/well) and subjected to the indicated treatments for 8 days. The cell colonies were fixed with 4% formaldehyde and stained with 0.1% crystal violet for 10 min, after which the colonies were counted. All assays were repeated three times.

### Cell migration assay

Cell migration was measured using a two-chamber Transwell migration assay as previously described. The lower chamber (24-well plate) was filled with 600 μL of DMEM-F12 medium containing 10% FBS. The upper chamber was filled with 200 μL of each group of medium prepared as described above, and 1 × 10^4^ HUVECs were seeded onto 8 μm pore size membranes (Selection 14341, Selection, Hefei, China). Unmigrated cells were swabbed from the upper chamber after 4, 8, 12, 24 and 48 h. The migrating cells were fixed with 4% paraformaldehyde and stained with crystal violet dye. The average number of migrating cells was counted using an inverted microscope at 100×.

### Scratch wound-healing assay

HUVECs (5 × 10^5^ cells/well) were inoculated in 6-well plates and incubated at 37 °C with 5% CO_2_. After the cells formed a fused monolayer, a sterile P1000 tip was used to produce a scratch in the center of the cell layer. The detached cells were gently rinsed off with PBS. Subsequently, the cells were treated with each of the media mixtures described above and imaged after 4, 8, 12, 24, and 48 h. ImageJ was used to estimate cell migration distances.

### Tube formation

The matrix gel (356234, BD-Pharmingen, USA) was diluted with DMEM-F12 at a 1:1 ratio and added to 48-well plates. The dishes were placed in an incubation chamber at 37 °C for 30 min to polymerize the matrix gel. HUVECs and HAECs (1 × 10^4^ cells/well) were resuspended in the various media mixtures described above, seeded into matrix gel-coated wells, and incubated in growth-supplement-free medium at 37 °C for 6 h. The samples were observed using an inverted microscope, and the tube formation was quantified using ImageJ.

### Establishment of animal models and assay validation

Eight-week-old male, 19–20 g purebred ApoE^−/−^ mice (*n* = 40, Beijing Vital River Laboratory Animal Technology Co., Ltd., China) were randomly divided into experimental (A–C) and control (D) groups of 10 mice each, and the experimental groups were randomly divided into three subgroups, including the A: MD-1 group (75 ng*0.072/g/mouse), calculated at 72 ml/kg of blood per mouse; B: MD-1&DNase I (50 μg/mouse) group; C: MD-1&TNP-470 (20 mg/kg, HY-101932, MedChemExpress, China) group; and D: Control group. All mice were housed in a specific pathogen-free facility and given weekly intraperitoneal injections for 16 weeks. A mouse atherosclerotic plaque model was generated using a Western diet containing 0.15% cholesterol and 21% fat (H10141, Beijing Huafukang Laboratory Animal Company, China). Assay procedures and protocols were reviewed and approved by the Animal Investigation Ethics Committee of the Second Affiliated Hospital of Harbin Medical University (YJSDW2022-116) and were performed in accordance with the Guidelines for the Care and Use of Laboratory Animals of the National Institutes of Health, China. At week 17, blood was taken from the apical part of the heart after sacrifice with excess sodium pentobarbital and centrifuged to retain the plasma, which was frozen and stored at −80 °C as extra samples. The carotid arteries were carefully isolated and cut and the obtained tissues were immersed in 4% paraformaldehyde overnight, paraffin-embedded, and divided into 4 μm paraffin sections for subsequent pathological staining, immunohistochemistry, and immunofluorescence assays (all as described previously).

### Depletion of neutrophils

We further confirmed that MD-1 plays an important role in regulating neutrophils. We constructed a neutrophil depletion model based on the in vivo injection of InVivoPlus anti-mouse Ly6G (BP0075-1-5MG, BioXCell, New Hampshire, USA). The MD-1 dose in both the Ly6G group and the MD-1 + Ly6G group was the same as that used previously, and the Ly6G dose was 5 μg/g/mouse/week (divided into three injections every other day) for 16 weeks. The feeding, collection, and experimental methods were the same as before.

### Flow cytometry

For the neutrophil depletion model group of mice, approximately 150 μl of peripheral blood was taken from the tail vein of mice and the erythrocytes were lysed, resuspended in cell staining buffer (420201), and incubated at room temperature with the addition of purified anti-mouse CD16/32 antibody (1.0 µg/10^6^ cells/100 µl, 101301) for 30 min. Then, each sample was incubated with FITC anti-mouse/human CD11b antibody (0.25 µg/10^6^ cells/100 µl, 101205) and APC anti-mouse Ly-6G antibody (0.06 µg/10^6^ cells/100 µl, 164506, BioLegend, San Diego, USA) for 40 min at room temperature protected from light. Finally, after washing and resuspension, the CD11b^+^ and Ly6G^+^ neutrophils in each sample were quantified via nanoflow analysis. The data were acquired using Apogee and analyzed using FlowJo X.

### Single-cell RNA-seq data analysis

Experimental single-cell data on carotid atherosclerotic plaques were obtained from the GSE155514 and GSE224273 datasets, and GSE131778 was used as the validation. The R package Seurat (ver. 5.0.1) was used to filter each single-cell sample and retain genes expressed in at least 3 cells and cells with at least 300 genes expressed. To reduce the effect of cell cycle genes, the cell cycle genes were scored in each cell, and the data were normalized. UMAP and t-SNE were used for cell clustering while significant differentially expressed genes in each cluster were calculated and the annotation of cell types was completed by CellMarker (http://xteam.xbio.top/CellMarker/index.jsp) and classical highly expressed genes common to all types of cells documented in the literature. Pseudotime analysis of the relevant cell types was performed using the R package Monocle 2. Intercellular communication analysis was performed using the R package CellChat to identify and predict interactions between different cell types. Transcription factor activity analysis was performed with the R package SCENIC.

### Statistical analysis

All the statistical analyses were performed using R software (ver. 4.1.3) or SPSS statistical software (ver. 22.0, IBM SPSS Inc., Chicago, IL, USA), or GraphPad Prism 9.0 (La Jolla, CA, USA). The data are shown as the mean ± standard deviation. One-way ANOVA followed by Tukey’s multiple comparison test was used for multiple groups. Student’s *t* test was used for comparisons between two groups. Significance levels are expressed as follows: ns *P* value ≥ 0.05, *P value < 0.05, **P value < 0.01, ***P value < 0.001, ****P value < 0.0001.

## Results

### Bioinformatics analysis of biomarkers associated with unstable carotid plaques

**Fig. 1:**
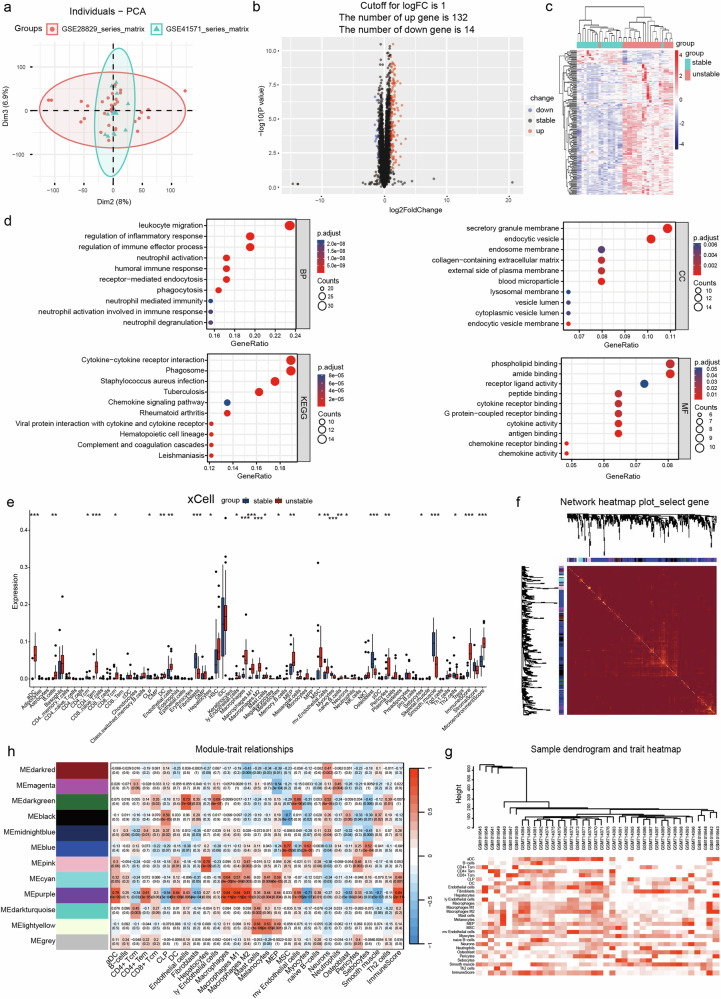
Screening of DEGs based on a coexpression network combined with immune infiltration correlation analysis. **a** PCA after combining the two datasets and removing batch effects. **b** Volcano plot. The X-axis represents log2 (fold change), the y-axis represents −log10 (*p* value), the red dots represent upregulated DEGs, and the blue dots represent downregulated DEGs. **c** Heatmap. The DEGs in each are in the corresponding column of the corresponding row of the samples and their expression levels are represented by color gradient: toward blue, the level is lower; toward red, it is higher. The classification tree on the left side depicts the similarity of the gene expression levels of the samples, where the more neighboring rows there are, the more similar the gene expression levels of the samples are in general. **d** GO and KEGG analyses. The gene ratio is a fraction, the numerator is the number of genes enriched in this GO enrichment, and the denominator is the number of all genes entered for enrichment analysis, which can be the number of genes obtained from differential expression analysis. p.adjust is the *P* value after correction. The Count value is the number of genes enriched in this GO term among the genes analyzed. **e** xCell analysis of 64 cell types. **f** WGCNA-based coexpression analysis of the TOM of 400 genes randomly selected from the module. **g** Sample clustering plot with a heatmap of sample traits representing the corresponding immune infiltration score for each sample. **h** Correlation plot of gene modules associated with sample traits related to immune infiltration.

PCA was used to cluster the samples after the two datasets were merged by debatching (Fig. 1a, Supplementary Fig. 1a–c). A total of 146 differentially expressed genes (DEGs) were identified by comparing the unstable and stable carotid plaque groups (Supplementary Table 3), of which 132 genes were upregulated and 14 were downregulated (Fig. 1b). Heatmaps were generated to visualize the gene expression patterns (Fig. 1c). GO analysis of differentially expressed genes was performed for genes with p.adjust < 0.05 and q.value < 0.2. According to the BP enrichment analysis, the differentially expressed genes were mainly involved in leukocyte wandering, regulatory effects on the inflammatory response, regulation of immune effector processes, and neutrophil activation. In the CC enrichment analysis, secretory granule membranes and intracellular vesicles were the most enriched CCs. The most enriched MF terms were anticipatory activity and phospholipid binding. In addition, we performed the Kyoto Encyclopedia of Genes and Genomes (KEGG) analysis to identify DEG-related pathways (Fig. 1d). Cytokine receptor interactions and vocal signaling pathways were significantly enriched. For this purpose, we quantitatively assessed the enrichment of 64 immune cells and stromal cells, cell types, and enrichment scores (Fig. 1e). The xCell scores of the stable and unstable plaque groups showed that the expression of genes characteristics of dendritic cells, ECs, macrophages, and pericytes was upregulated in the unstable plaque group. In contrast, the expression of genes characteristic of fibroblasts and smooth muscle cells was upregulated in the stable plaque group. The difference between the total immunity score and the microenvironment score indicates the presence of immune infiltration in unstable plaques. No outliers were excluded under hierarchical clustering, and a scale-free network was constructed to satisfy the scale-free topology using a soft threshold of *β* = 8 and a correlation coefficient threshold of 0.85 (Supplementary Fig. 1d, e). Then, a topology overlap matrix (TOM) was created, and 400 genes were randomly selected from the module (Fig. 1f). The dark red color indicates a strong degree of overlap between these genes. To identify key modules associated with sample immune infiltration traits, we plotted sample clusters with sample trait heatmaps by organizing immune infiltration-related data and merged clustering graphs (Fig. 1g, Supplementary Fig. 1f). Gene modules that were correlated with immune cell infiltration were plotted (Fig. 1h). Key modules with significant (*P* < 0.05) and robust correlations (cor > 0.8) with immune infiltration-related modules (Supplementary Fig. 1g), such as the purple module, were selected for additional analysis.

### Selection and identification of hub genes

**Fig. 2:**
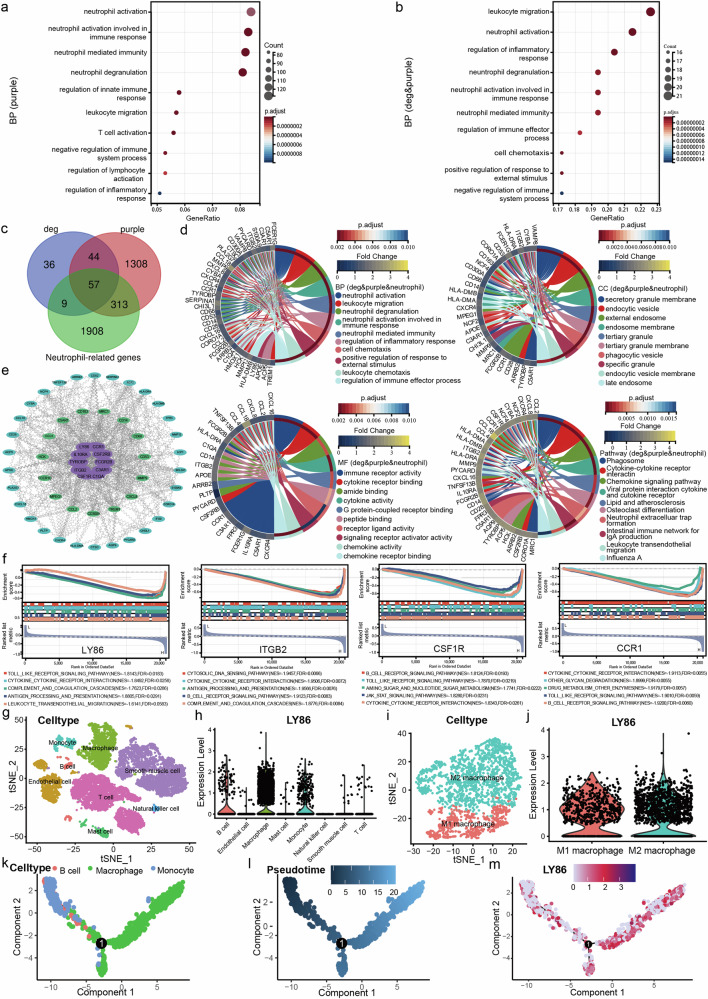
Enrichment analysis and screening of hub genes using key modular rows associated with sample traits. **a** GO (BP) enrichment analysis of purple module genes. **b** GO (BP) enrichment analysis of shared DEGs and purple module genes. **c**, **d** GO and KEGG enrichment analysis of the shared DEGs, purple module genes, and neutrophil-related genes. **e** PPI network construction and selection of hub genes, where core modular genes of the MCODE screen are located in the middle and inner circles, and hub genes of the cytoHubba screen are located in the inner circle. **f** GSEA was used for the final screening of hub genes. NES represents the average of the enrichment scores obtained for each pathway, and FDR determines the rate of false-positive findings that may be contained therein. Absolute NES values were negatively correlated with FDR, indicating high enrichment and reliable results. **g** t-SNE visualization showing the outcomes of cell type annotation. The expression levels of all genes expressed by cells in each cluster were quantified and ranked, followed by the annotation of cell types using various methods. **h** The expression of LY86 in various cell types was examined using single-cell transcriptome data associated with the formation of unstable carotid plaques. **i** Macrophage subpopulations. **j** Expression of LY86 in different subpopulations. **k** Presentation of the three cell types in the pseudotime analysis trajectory. **l** Pseudotime order in the pseudotime analysis trajectory. **m** The differentiation of LY86 in the pseudotime analysis trajectory.

We identified 1723 genes from the purple module (Supplementary Table 4), performed a GO enrichment analysis, studied the BPs of the key genes associated with the sample traits (Fig. 2a), and showed that these genes are involved mainly in neutrophil activation, neutrophil activation involved in the immune response, neutrophil-mediated immunity, neutrophil degranulation, and so on. We further compared the purple module genes with the DEGs and used a total of 101 genes (Supplementary Table 5) for BP analysis (Fig. 2b); these genes are involved mainly in leukocyte wandering, neutrophil activation, inflammatory response regulation, neutrophil degranulation and so on. A total of 57 shared genes (Fig. 2c) identified by comparing the DEGs and purple module genes and neutrophil-related genes (Supplementary Table 6) were again subjected to GO and KEGG analyses (Fig. 2d), and the BP enrichment analysis showed that these genes were involved mainly in neutrophil activation and leukocyte wandering. The CC term secretion of granulose membranes was enriched mainly in these genes MF enrichment analysis showed that these genes were involved mainly in immune receptor activity and cytokine receptor binding. KEGG showed that these genes were involved primarily in cytokine receptor interactions, lipid and AS, and neutrophil extracellular trap formation. To determine the interactions among these 57 genes, we constructed a PPI network using STRING according to the library and visualized it with Cytoscape software (Fig. 2e). The top 10 genes (FCGR2B, TYROBP, CCR1, ITGB2, C3AR1, IL10RA, LY86, C1QA, CSF2RB and CSF1R) were screened from the core module based on the MCC algorithm of MCODE and cytoHubba. ROC curve analysis was subsequently performed to determine the sensitivity and specificity of the hub genes in the diagnosis of unstable plaques by comparing the area under the curve (AUC) values. In the experimental dataset, eight hub genes had AUC values > 0.92, and two hub genes had AUC values > 0.86, indicating that these genes are highly valuable for the diagnosis of unstable carotid atherosclerotic plaques (Supplementary Fig. 2a). To improve the reliability of the results, we used an external dataset (GSE43292) to validate the expression levels and receiver operating characteristic (ROC) curves of these 10 hub genes (Supplementary Fig. 2b, c). Four genes were further screened by logFC and UniProt-related information from the validation set, including CCR1, ITGB2, LY86, and CSF1R (Table 1). GSEA was used to characterize the four hub genes in the gene set (Fig. 2f). Among these genes, the LY86 gene, also known as MD-1, is the only gene that secretes a protein and is closely related to the TLR4 gene. A comprehensive analysis of the previous literature and the abovementioned bioinformatics analysis was performed to determine whether MD-1 (*LY86*) might further activate NETs through TLR4 and thus cause carotid plaque instability.

**Table 1:** Functional profiles, AUC, and subcellular localization of the hub genes.

Gene Symbol	Function	AUC	Subcellular localization
MD-1 (*LY86*) (Myeloid differentiation protein-1, *Lymphocyte antigen 86*)	May synergize with RP105 and TLR4 to mediate innate immune responses to bacterial LPS and cytokine production. Important for effective RP105 cell surface expression^39^.	0.93	Secreted; Extracellular space
CSF1R (Colony-stimulating factor 1 receptor)	Promotes the release of pro-inflammatory chemokines in response to IL34 and CSF1 stimulation, thus playing an essential role in intrinsic immunity and inflammatory processes^40^. Promotes actin cytoskeleton reorganization, regulates membrane ruffling and cell adhesion and cell migration, and promotes cancer cell invasion^41^.	0.92	Cell membrane
ITGB2 (Integrin beta-2)	Integrin ITGAM/ITGB2 is also a receptor for factor x. Integrin ITGAD/ITGB2 is a receptor for ICAM3 and VCAM1. Contributes to natural killer cell cytotoxicity^42^. Involved in the adhesion and migration of leukocytes including T cells and neutrophils^43^. Triggers neutrophil migration during lung injury through PTK2B/pyk2-mediated activation^44^. The integrin ITGAL/ITGB2 binds to ICAM3 and promotes phagocytosis of apoptotic neutrophils by macrophages^45^.	0.94	Cell membrane; Membrane rafts
CCR1 (CC-chemokine receptor type 1)	A chemokine receptor of type C-C. Binds to mip-1α, mip-1δ, RANTES, and MCP-3, and less efficiently to mip-1β or MCP-1, and subsequently transduces signals by increasing intracellular calcium levels. Regulates stem cell proliferation^46^.	0.94	Cell membrane

### Validation and exploration of LY86 and other key genes involved in carotid-unstable plaque formation based on single-cell transcriptome sequencing data

To further explore the genes associated with unstable carotid plaques, we collected scRNA-seq data on carotid plaques. After quality control and other screening (Supplementary Fig. 3), we obtained 10 cell clusters (Supplementary Fig. 4a, b). After annotation, 8 cell types closely related to unstable carotid plaques, such as macrophages and monocytes, were finally obtained (Fig. 2g, Supplementary Fig. 4c), and the validation of the cell type annotation was completed using the highly expressed genes of each type (Supplementary Fig. 4d) and the classical cell type markers (Supplementary Fig. 4e). According to the integrated single-cell sequencing of differentially expressed genes, LY86 was significantly more highly expressed in macrophages and relatively more highly expressed in monocytes and B cells (Fig. 2h). We extracted the full range of macrophage marker genes, performed subpopulation analysis and clustering and annotated the genes with high expression in each cluster, ultimately splitting them into M1 macrophages and M2 macrophages (Fig. 2i). The percentage of M1 macrophages expressing LY86 was significantly greater than in the percentage of M2 macrophages expressing LY86 (Fig. 2j). We performed a similar analysis on the GSE131778 validation set and obtained consistent results (Supplementary Fig. 4f). We conducted pseudotime analysis on LY86-enriched cell populations, including macrophages, monocytes, and B cells. As pseudotime progressed, monocytes gradually differentiated into macrophages, mirroring the differentiation process observed in macrophages (Fig. 2k, l). Moreover, the expression of LY86 gradually increased along the trajectory of pseudotime differentiation (Fig. 2m). GO enrichment analyses of the top 20 highly expressed genes in the three abundant cell populations were performed and integrated into the high-expressing LY86 cell types for summarization (Supplementary Fig. 5). To further elucidate the underlying mechanism governing the LY86 differentiation trajectory, we conducted cell communication analysis to investigate the interactions, potential targets, and signaling pathways among diverse cell types during LY86 differentiation (Supplementary Figure 6a, b). Based on the findings from the pseudotime analyses, we identified the communication networks established between distinct cell types, where monocytes served as initiators and macrophages acted as receivers (Supplementary Fig. 6c). The investigation of the LY86 upstream regulatory network was predominantly focused on transcription factors (Supplementary Fig. 7, Supplementary Table 7).

### A significant increase in gene expression was observed in unstable carotid plaques

**Fig. 3:**
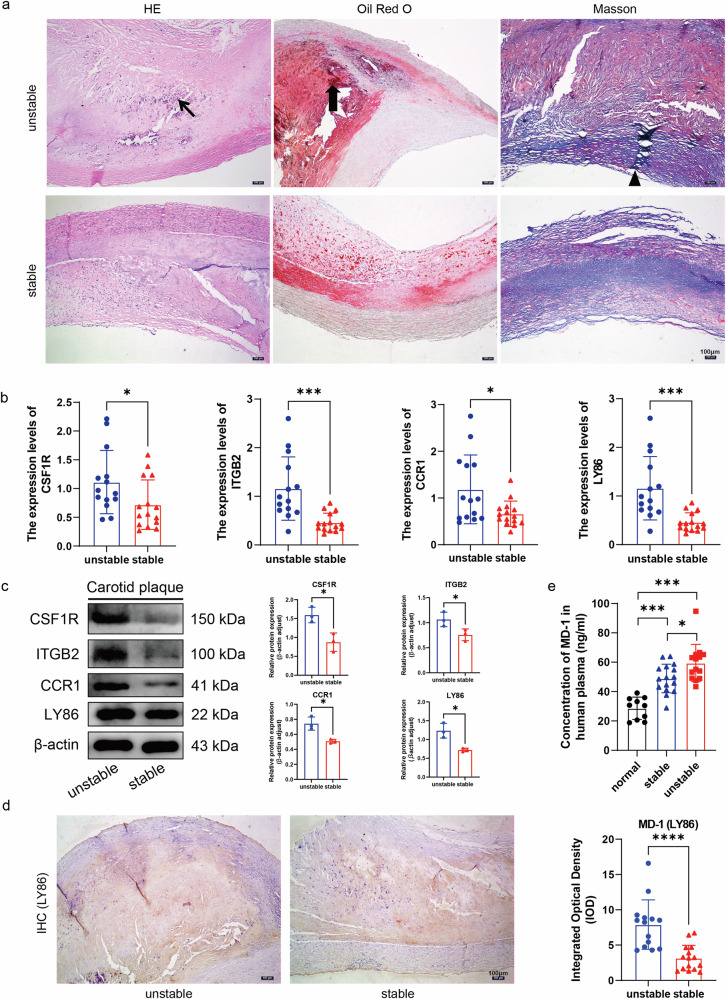
Pathologic staining and validation of carotid plaques. **a** HE staining, oil red O staining, and Masson trichrome staining (40× magnification, scale bar = 100 μm). Thin black arrows indicate intraplaque hemorrhage, thick black arrows indicate large lipid cores and black triangles indicate discontinuous fibrous caps. **b** RT‒qPCR results of the four hub genes in the stable and unstable plaque groups. **c** Western blot results of four hub genes in the unstable and stable plaque groups. β-Actin was used as an internal control. **d** Immunohistochemical staining was used to quantify the total amount of MD-1 (*LY86*, yellowish brown, scale bar = 100 μm) within the plaques. **e** Concentration of MD-1 in plasma in the healthy volunteer group and the stable and unstable plaque patient groups.

The clinical characteristics of the patients were analyzed by retrospectively assessing sex, age, hypertension status, hyperlipidemia status, coronary artery disease status, diabetes mellitus status, and kidney disease status. There was no significant difference in any of the clinical characteristics between the two groups (Supplementary Table 8). Carotid plaque pathology (Fig. 3a) and HE staining revealed that the atherosclerotic plaque area in the unstable plaque group was greater than that in the stable plaque group, and intraplaque hemorrhage was detected. Atherosclerotic plaques, aortic intimal thickening, and an increase in the amount of oil-red O-stained lipids were observed in the unstable plaque group. Masson’s trichrome staining of the unstable plaque group revealed an uneven thickness of the fibrous cap of the atherosclerotic plaques, poor continuity and marked thinning, and a decrease in the content of collagen fibers. Twenty-nine patients who underwent carotid endarterectomy were enrolled in the study, and the patients were divided into stable and unstable plaque groups based on macroscopic and histological data. The RT‒qPCR results (Fig. 3b) showed that the expression of LY86, CSF1R, ITGB2, and CCR1 was significantly upregulated in the unstable plaque group, which was in agreement with our bioinformatics analysis. The protein expression levels of MD-1 (*LY86*), CSF1R, ITGB2, and CCR1 in plaque tissues (Fig. 3c) were greater in the unstable plaque group than in the stable plaque group, which was consistent with our bioinformatics analysis and RT‒qPCR results. Immunohistochemistry was used to quantify MD-1 within 29 carotid plaques (Fig. 3d). The expression was greater in the unstable plaque group than in the stable plaque group. The difference in staining between the two groups was statistically significant. This finding is in agreement with our bioinformatics analysis, RT‒qPCR, and Western blot results. The concentration of MD-1 in the plasma of 30 patients and 10 healthy volunteers was measured by ELISA (Fig. 3e), of which 14 had histologically unstable plaques and 16 had stable plaques. Patients in the unstable plaque group had significantly greater plasma concentrations of MD-1 than those in the stable plaque group and significantly greater plasma concentrations than those in the healthy volunteer group.

### MD-1 induces NETs formation

**Fig. 4:**
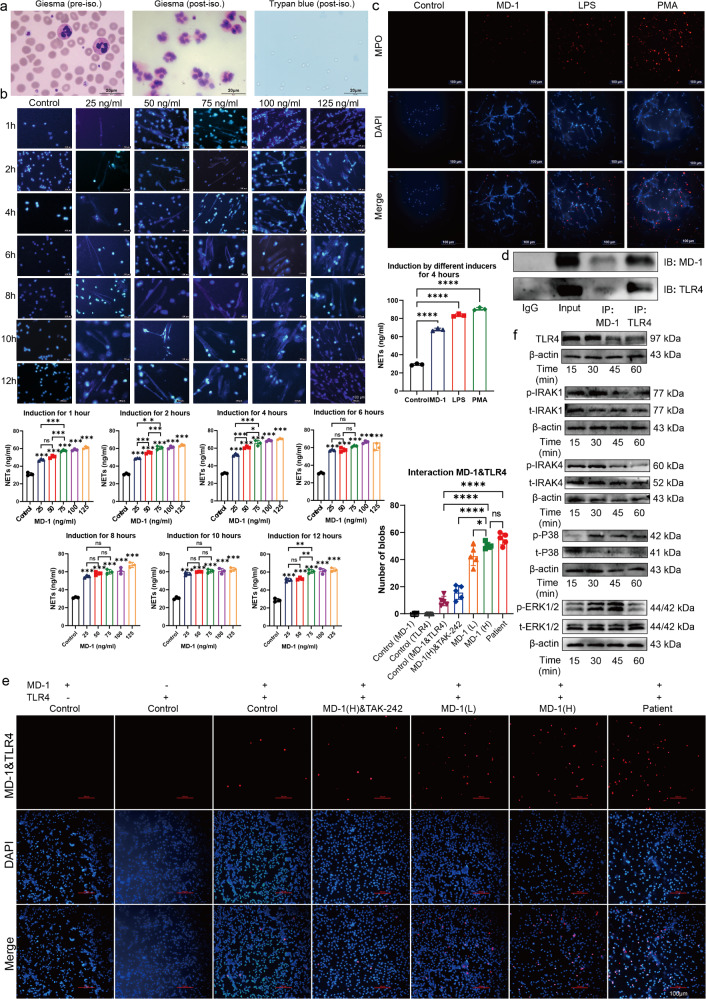
MD-1 induces the formation of NETs. **a** Isolation and validation of neutrophils (Giemsa staining, Trypan blue staining, and cell counter, scale bar = 20 μm). **b** Hochest staining and ELISA were used to quantify the concentration and time course of MD-1-induced NETs. **c** NETs were quantified in the negative control group, the MD-1 (75 ng/ml) group, and the two positive control groups. **d** Co-IP revealed that MD-1 and TLR4 coexisted on neutrophils. **e** The interaction between MD-1 and TLR4 in peripheral blood neutrophils from carotid plaque patients and peripheral blood neutrophils from healthy volunteers induced under different conditions was analyzed by Duolink PLA (scale bar = 100 μm). **f** Western blot analysis showed that neutrophils induced with MD-1 upregulated TLR4 expression and increased the phosphorylation of IRAK1/IRAK4, p38 MAPK, and ERK1/2 in a time-dependent manner (from 0 to 60 min). t total value; p phosphorylation value. β-Actin was used as an internal control.

Neutrophils were isolated from the peripheral blood of healthy volunteers and identified (Fig. 4a). Giemsa staining was used to assess the purity of isolated neutrophils >97% and Trypan blue staining was used to assess the viability of neutrophils. A representative image shows that the viability ranged 82.1–97.7%, according to the cytometer data. To investigate the effect of MD-1 on NETs formation, we treated neutrophils with MD-1 at different concentrations and for different durations (Fig. 4b) and positive controls (Fig. 4c), where 25 ng/ml was considered the in vivo concentration in healthy individuals, 50 ng/ml was the in vivo concentration in patients with stable plaques, 75 ng/ml was the in vivo concentration in patients with unstable plaques, and the remaining concentrations (100 ng/ml, 125 ng/ml) were the experimental design concentrations. There were more NETs in every group than in the control group after 1 h of induction, with statistically significant differences between the 25 ng/ml, 50 ng/ml, and 75 ng/ml groups after 2 h and 4 h of induction. There was no statistically significant difference between the above three concentration groups at 6, 8, 10, and 12 h, and the total amount of NETs produced by the same concentration group was not significantly greater after 6 h of induction. Hoechst staining was conducted to quantify the NETs produced at different concentrations at the same time of induction, and the formation of NETs was by the protrusion of reticulated DNA filaments. The results showed that the formation of NETs induced by MD-1 is concentration- and time-dependent. Therefore, a concentration of 75 ng/ml and an induction time of 4 h were used in the subsequent study, as this concentration is more consistent with the results measured in human peripheral blood in the above assays and is similar to those described in the literature on NETs. Previous studies^12^ have shown that MD-1 can interact with TLR4 on neurons and macrophages, whereas the interaction of MD-1, which is a secreted protein in the blood, with TLR4 on the surface of neutrophils has not been reported. For this purpose, we investigated the role of TLR4 in the formation of MD-1-induced NETs. We performed a co-IP assay (Fig. 4d) and found that MD-1 co-precipitated with TLR4 on neutrophils by pulling TLR4 co-precipitated with MD-1 and MD-1 co-precipitated with TLR4 MD-1 coprecipitated with TLR4 on neutrophils by pulling down TLR4, which coprecipitated with both MD-1 and MD-1. Subsequently, a Duolink proximity ligation assay was employed to validate the findings obtained through co-IP (Fig. 4e). We designated MD-1(L) as MD-1 at a concentration of 50 ng/ml in the stable plaque group and MD-1(H) as MD-1 at a concentration of 75 ng/ml in the unstable plaque group and referred to the group with additional TLR4 inhibitors as MD-1(H) and TAK-242. Quantitative analysis revealed that the quantification of the PLA signal on neutrophil surfaces in patients with unstable carotid artery plaques was comparable to that of patients with unstable carotid plaques treated with MD-1, further validating the feasibility of utilizing MD-1 (75 ng/ml) in in vitro experiments as a representative concentration for MD-1 in the unstable plaque group in vivo. Furthermore, the MD-1(L), MD-1(H), and TAK-242 groups exhibited greater levels of PLA than the healthy volunteer group. Negative controls were established by employing either an anti-MD-1 antibody or an anti-TLR4 antibody alone. In conclusion, our Duolink PLA experiments provided further evidence that secreted MD-1 specifically binds to TLR4 receptors expressed on the surface of human peripheral blood neutrophils. Western blot analysis (Fig. 4f) revealed that MD-1 promoted the time-dependent phosphorylation of IRAK1/IRAK4, p38 MAPK, and ERK1/2 in neutrophils.

### Activation of the Toll-like receptor signaling pathway is required for MD-1-induced NETs formation

**Fig. 5:**
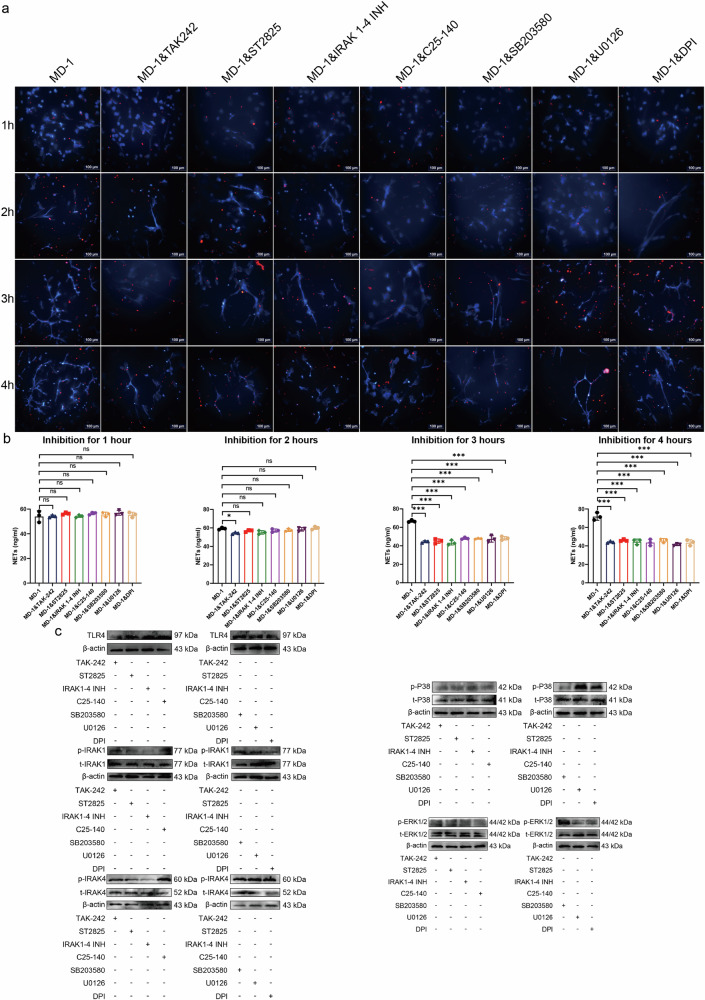
MD-1 induces the formation of NETs via the Toll-like receptor pathway. **a**, **b** Probing the temporal gradient of induction of NETs upon addition of pathway inhibitors (scale bar = 100 μm). **c** Inhibition of TLR4, MyD88, IRAK1-4, and TRAF6 with specific inhibitors abrogated MD-1-induced TLR4 expression as well as the phosphorylation of IRAK1/IRAK4, p38 MAPK, and ERK1/2; inhibition of NADPH oxidase with DPI did not affect the phosphorylation of p38 MAPK or ERK1/2. t total value; p phosphorylation value. β-Actin was used as an internal control.

Each set of the eight immunofluorescence plots corresponds to the quantification of NETs after 75 ng/ml MD-1 induction and after the addition of seven different pathway inhibitors. The specific structure of NETs was determined by the prominence of reticulated DNA filaments (blue) and the quantification of the cytoplasmic granule protein MPO (red) performed by software (Fig. 5a, b). There was no significant difference in the quantification of NETs after 1 h of induction with MD-1; moreover, among the groups treated with different pathway inhibitors, only the TAK-242 (a TLR4 inhibitor) group showed a statistically significant decrease in the number of NETs at 2 h of induction, and the number of NETs was significantly decreased in all groups after 3 and 4 h of induction, with the number of NETs in the 4-hour group being most significantly decreased. Therefore, 4 h after the addition of the inhibitor was chosen as the time point for subsequent assays. First, neutrophils were treated with 1 μM TAK-242 (a TLR4 inhibitor), 10 μM ST2825 (a MyD88 inhibitor), 0.5 μM IRAK1-4 inhibitor (an IRAK1 and an IRAK4 inhibitor), 10 μM C25-140 (a TRAF6 inhibitor), 10 μM SB203580 (a p38 MAPK inhibitor), 10 μM U0126 (an ERK1/2 inhibitor) or 10 μM DPI (an NADPH inhibitor) before MD-1 (75 ng/mL) was added to neutrophils, and proteins were extracted for subsequent assays. As shown in Fig. 5c, the phosphorylation of IRAK1/IRAK4, p38 MAPK, and ERK1/2 was abrogated by the TAK-242, ST2825, or IRAK1-4 inhibitor. The C25-140 inhibitor also abrogated p38 MAPK and ERK1/2 phosphorylation. The phosphorylation of p38 MAPK was reduced by the SB203580 inhibitor, and the phosphorylation of ERK1/2 was suppressed by the U0126 inhibitor, suggesting that MAPKs are downstream signaling molecules of the TLR4/MyD88/IRAK pathway. In summary, our results suggest that the Toll-like receptor signaling pathway is an intracellular signaling pathway that is critical for the formation of NETs induced by MD-1.

### MD-1-induced NETs promote the proliferation of HUVECs but play an unfavorable role in migration

**Fig. 6:**
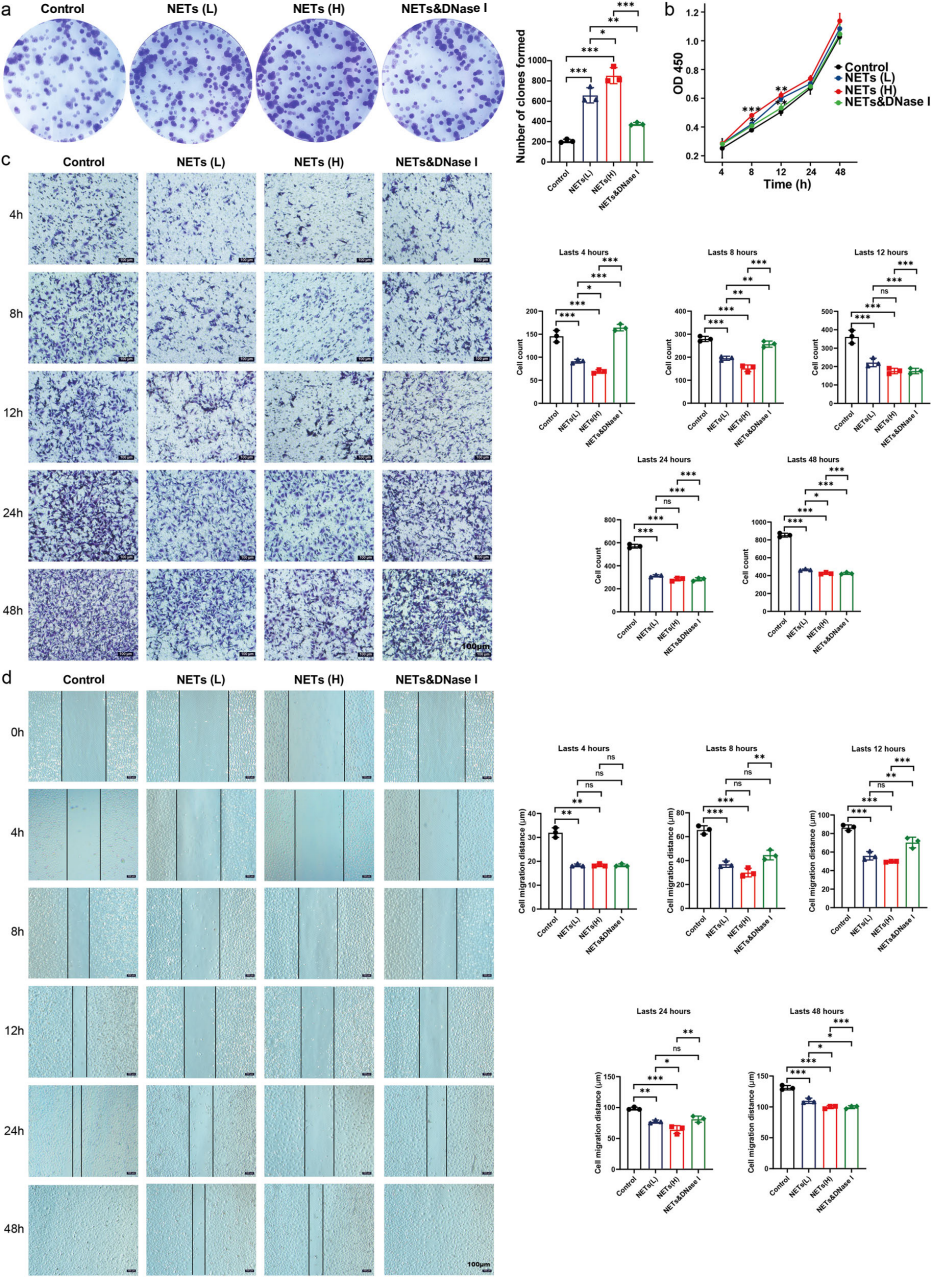
NETs promote the proliferation of HUVECs while inhibiting their migration. **a** HUVECs treated with ddH_2_O and DMSO (control), a low concentration of NETs (NETs (L)), an elevated concentration of NETs (NETs (H)), or NETs combined with DNase I (NETs&DNase I) were subjected to a colony formation assay. **b** HUVECs were treated in the same way and subjected to a CCK-8 assay. **c** Transwell migration assay of HUVECs treated in the same way. **d** Scratch healing assay of HUVECs treated in the same way (Scale bar = 100 μm).

Vascular ECs play a decisive role in the maintenance of intravascular homeostasis, and intraplaque neovascularization is significantly affected by NETs, which are therefore essential for maintaining carotid plaque stability. To explore the potential effect of NETs on carotid plaque progression, we performed a CCK-8 assay and found that NETs promoted the proliferation of HUVECs, and this effect was more pronounced at higher concentrations, whereas this effect was abolished in the NETs and DNase I groups (Fig. 6b). Consistent with this effect, the cell migration in the groups stimulated with NETs was greater than that in the control group in the colony formation assay (Fig. 6a). Transwell assays revealed that NETs inhibited HUVECs migration and that the inhibitory effect was greater in the high-concentration group than in the low-concentration group, while this effect was eliminated in the NETs and DNase I group (Fig. 6c). Consistent with this effect, the cell migration distance the groups stimulated with NETs was lower than that in the control group in the scratch assay (Fig. 6d).

### NETs activate the NF-κB signaling pathway and increase the expression of VCAM1, ICAM1, MMP14, VEGFA, and IL6 in ECs to influence the plaque microenvironment and thus promote neovascularization within the plaque

**Fig. 7:**
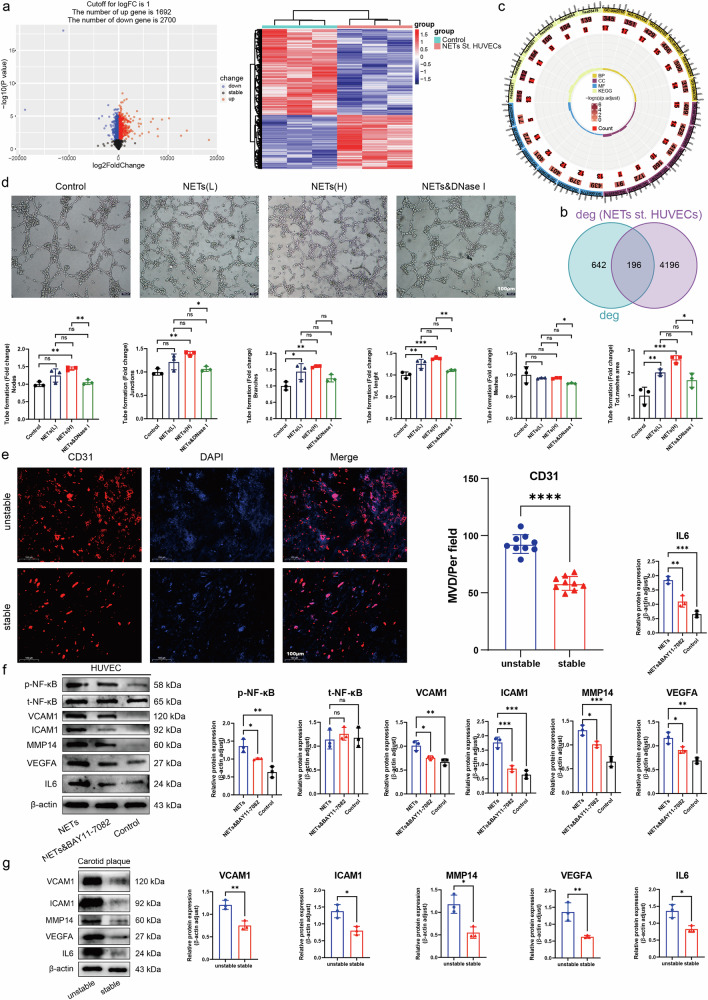
NETs activate the NF-κB signaling pathway to promote intraplaque neovascularization. **a** HUVECs were screened for relevant DEGs after NETs stimulation, and the results were visualized in the form of a volcano plot and heatmap. **b** Intersection of deg and deg (NETs st. HUVEC) related genes. **c** GO and KEGG analyses. **d** Tube formation assay with HUVECs treated as described above. **e** Representative images of immunofluorescence staining visible in unstable plaques of the middle carotid artery and within stable plaques with CD31 (red) showing intraplaque neovascularization. The MVD was significantly different between the two groups. (Scale bar = 100 μm). **f** Western blot showing the expression of VCAM1, ICAM1, MMP14, p-NF-κB, t-NF-κB, VEGFA, and IL6 in HUVECs under different conditions. **g** Expression of VCAM1, ICAM1, MMP14, VEGFA and IL6 in tissues. t total value; p phosphorylation value. β-Actin was used as an internal control.

To further explore the influence of NETs on the stability of carotid plaques through their effect on ECs, we again performed relevant bioinformatics analyses (Fig. 7a, Supplementary Table 9). The DEGs from this dataset were compared with the above carotid plaque-related DEGs (Fig. 7b), and further, GO and KEGG enrichment analyses were performed on the shared DEGs. The three terms with the highest enrichment are presented. The BP terms regulation of angiogenesis and regulation of vasculature system were highly enriched. KEGG analysis revealed that the TNF signaling pathway, pathways associated with lipid and AS, and the NF-κB signaling pathway were enriched (Fig. 7c). By summarizing the results of differential gene expression analysis and enrichment analysis and analyzing the related literature, we argue that NETs may promote neovascularization within the plaque, thereby destabilizing the carotid plaque. Therefore, we assessed the effect of NETs on the angiogenic capacity of HUVECs and HAECs by tube-forming assays. Exposure to NETs increased the formation of tubes, however, they were not fully formed, which was supported by the results of the nodes, junctions, branches, total length, and grid area of the tubes formed by high concentrations of NETs. These values were higher than those in the group treated with low concentrations of NETs and the control group in turn, but the number of grids with intact tubes was indeed the same as that of the other groups or slightly lower, which meant that the number of incomplete tubes was greater than that of the other groups (Fig. 7d). The same outcomes were achieved when HAECs were used for tube formation experiments (Supplementary Fig. 8a). To further explore the underlying mechanism, we selected gene that was highly correlated with tube formation, namely, VCAM1, ICAM1, MMP14, VEGFA, and IL6 (Supplementary Table 10), for subsequent validation and considered that the expression of the abovementioned genes might be upregulated through activation of the NF-κB signaling pathway. To determine whether the formation of unstable plaques was associated with intraplaque neovascularization, we performed immunofluorescence staining of carotid plaque tissues from both the unstable and stable plaque groups. The results showed that the expression of CD31 was significantly greater in the unstable plaque group than in the stable plaque group, and there was a statistically significant difference in the density of the microvessels (Fig. 7e). Western blot results showed that NETs promoted the phosphorylation of NF-κB in HUVECs, and the expression of its related downstream signaling molecules, VCAM1, ICAM1, MMP14, VEGFA and IL6, was significantly greater than that in the control group. We treated HUVECs with 5 μM BAY11-7082 (an NF-κB inhibitor). These inhibitors significantly reduced the phosphorylation level of NF-κB, and the expression of downstream signaling molecules was significantly lower than that in the NETs group. This indicated that NETs were involved in the activation of the NF-κB signaling pathway (Fig. 7f). We conducted identical validation experiments using HAECs, which yielded consistent Western blot analysis results (Supplementary Fig. 8b). To further verify whether the aforementioned downstream molecules are associated with unstable plaque formation, we examined unstable plaque tissue in the carotid artery versus stable plaque tissue. Western blot results showed that the expression of VCAM1, ICAM1, MMP14, VEGFA, and IL6 was significantly greater in the unstable plaque group than in the stable plaque group (Fig. 7g). In conclusion, the results of the experiments using both cell types and the bioinformatics analysis showed that the NF-κB signaling pathway is a crucial intracellular signaling pathway through which NETs increases neovascularization in plaques.

### MD-1 induced the formation of NETs, which subsequently increased neovascularization within the carotid plaques of mice, thereby destabilizing carotid plaques in an animal model

**Fig. 8:**
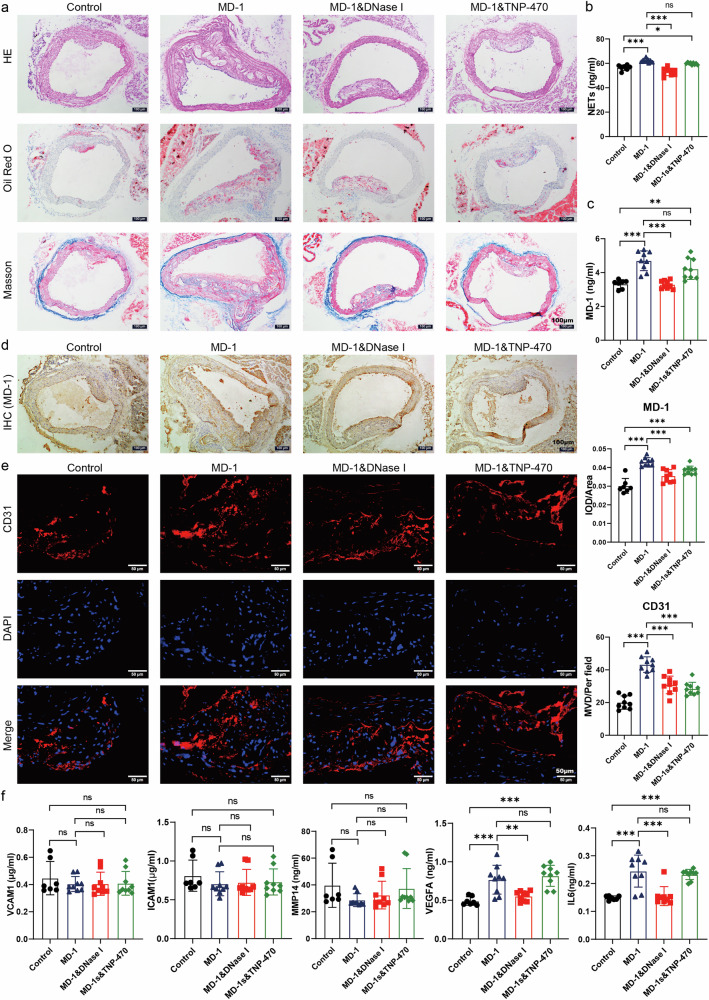
Influence of MD-1-induced NETs production on atherosclerotic unstable plaque formation in ApoE^-/-^ mice. **a** Representative images of carotid plaques from each group of mice stained with HE, oil red O, and Masson trichrome to evaluate the stability of carotid plaques (scale bar = 100 μm). **b** The levels of NETs in the plasma of mice in each group were measured by ELISA. **c** Quantification of MD-1 within the plaques of mice in each group by immunohistochemical staining and representative images of each group (scale bar = 100 μm). **d** Quantification of MD-1 in the plasma of mice in each group was performed by ELISA. **e** Immunofluorescence staining for CD31 (red) showing representative images of neovascularization conditions within the plaques of mice and the calculated MVD in each group (scale bar = 50 μm). **f** Expression levels of VCAM1, ICAM1, MMP14, VEGFA, and IL6 in the plasma of mice in each group were measured by ELISA.

To investigate the regulatory effect of MD-1 on the formation of NETs in a mouse model, the levels of NETs in the plasma of each group of mice were measured by ELISA. The expression levels of NETs were significantly greater in the MD-1 group and the MD-1&TNP-470 group than in the MD-1&DNase I group and the control group (Fig. 8b). To explore the effect of MD-1-induced NETs production on carotid plaques, we performed HE, oil red O, and Masson trichrome staining on mouse carotid plaques to assess plaque stability. We found that HE staining revealed diffuse thickening of the carotid intima in all groups of mice, all of which formed typical atherosclerotic plaques, and that the size of the carotid plaque deposits was significantly greater in the MD-1 group than in the other groups. Compared with those in the MD-1 group, the size of the plaques in the MD-1&DNase I and MD-1&TNP-470 groups significantly decreased. In addition, Masson trichrome and oil red O staining showed that the plaques in the MD-1 group had large amounts of branched cholesterol crystals, thinner fibrous caps that showed discontinuities, and larger lipid pools. Compared to the MD-1 group, the MD-1&DNase I group and the MD-1&TNP-470 group had significantly reduced lipid-stained areas and more continuous fiber caps; therefore, it can be inferred that TNP-470 inhibited the number of neovascular structures in plaques during plaque development (Fig. 8a). However, immunohistochemical staining showed that the quantification of MD-1 within the plaques of mice in the MD-1 group and MD-1&TNP-470 group was greater than that in the other groups (Fig. 8c). Therefore, we wondered whether NETs further increase MD-1 levels in vivo. Moreover, the amount of MD-1 in the plasma of each group measured by ELISA was consistent with the immunohistochemical results (Fig. 8d). Flow cytometry analysis revealed a significant depletion of neutrophils in the peripheral blood of mice across all experimental groups following the administration of the neutralizing antibody. Furthermore, consistent observations at multiple time points confirmed a sustained and continuous reduction in neutrophil levels within the mouse model (Supplementary Fig. 9a). Compared with those in the MD-1 group in the experimental cohort, all groups in the neutrophil depletion model group exhibited a significant reduction in carotid plaque deposition. Additionally, oil red O staining revealed significantly lower lipid pool levels than those in the MD-1 group, and Masson staining demonstrated more continuous fibrous cap formation. However, intragroup analysis within the neutrophil depletion model indicated no significant difference in these staining results between the two groups. This finding suggested that neutrophil depletion effectively reversed the impact of MD-1 on unstable plaque production in carotid arteries (Supplementary Fig. 9b). Furthermore, there was no notable difference in the quantitative MD-1 results through immunohistochemistry between the two groups in the neutrophil depletion model; however, these outcomes were lower than those observed in the control group (Supplementary Fig. 9c). Considering that neutrophil depletion could influence NETs formation and subsequently affect MD-1 levels, it can be further hypothesized that NETs may induce an additional increase in vivo. We further investigated the effect of NETs on intraplaque neovascularization in mice. Immunofluorescence staining revealed a significant increase in intraplaque neovascularization in the MD-1 group compared with the remaining groups (Fig. 8e), and there was also a statistically significant difference in the MVD. The results showed that NETs significantly increased intraplaque neovascularization. To investigate whether intraplaque neovascularization in mice is related to the plaque microenvironment, we used ELISA to measure the expression levels of VCAM1, ICAM1, MMP14, VEGFA, and IL6 in mouse plasma. The expression levels of VCAM1, ICAM1, and MMP14 were not significantly different among the groups, while the expression levels of VEGFA and IL6 in the plasma of mice in the MD-1 group and MD-1&TNP-470 group were significantly greater than those in the control group; therefore, NETs stimulated an increase in the expression levels of VEGFA and IL6 in the plasma, and this effect could still be inhibited by DNase I (Fig. 8f). In summary, MD-1 induces the formation of NETs, alters the plaque microenvironment, increases intraplaque neovascularization, and promotes the formation of unstable carotid plaques in mice.

## Discussion

Identifying biomarkers and possible therapeutic targets of unstable carotid plaques in patients at risk of stroke is essential for the prevention of adverse cerebrovascular and cardiovascular events^20^. The genes identified in this study, which are associated with carotid-unstable plaques, are predicted to be involved in BPs such as neutrophil activation and immune responses. This suggests that the development of unstable plaques is a complex process regulated by an immune-inflammatory response. This possibility is consistent with evidence that plaque instability is primarily triggered by unresolved inflammatory responses^21^.

We identified a diverse array of cells within carotid plaques that exhibit intricate intercellular interactions, thereby establishing a distinctive microenvironment. Consequently, it is imperative to identify and target pivotal genes that foster close cellular associations. In conjunction with prior investigations, the findings of this study indicate that these genes not only directly impact macrophage and EC function through their influence on macrophages but also further contribute to endothelial dysfunction by modulating neutrophils, thereby promoting carotid plaque instability. This disruption of endothelium-mediated vasoconstrictive and diastolic responses involves inflammatory responses, immune function, and oxidative stress^22^. Neutrophils play an active role in the early stages of AS, contributing to the amplification of local inflammation and tissue damage through their recruitment of other immune cells^23^. Among the potential outcomes of neutrophil activation, a specific cellular death process known as NETs formation has been identified^24^. Notably, neutrophils primarily adhere to atherosclerotic plaques through the mechanism of NETs formation^8^. Consequently, NETs directly interact with ECs and contribute to endothelial barrier damage and increased permeability^16^. The protein MD-1 (*LY86*) is a secreted molecule that is closely associated with the Toll-like receptor signaling pathway. Neutrophils express most members of the TLR family, including TLR4, and activation of cell surface TLRs has been shown to play multiple roles, including cytokine production^9^. Therefore, we propose for the first time that high concentrations of MD-1 in plasma may bind to the TLR4 receptor on the surface of neutrophils, further inducing the production of NETs through activation of the Toll-like receptor signaling pathway, which in turn affects plaque stability. Previous studies have demonstrated the essential role of MD-1 in facilitating cell surface expression and interaction with the extracellular structural domain of RP105^10^. Furthermore, this complex comprising MD-1 and RP105 has been implicated in the regulation of vascular remodeling and atherosclerotic plaque development; however, it predominantly exerts inhibitory effects on TLR signaling pathways, including the TLR4 signaling pathway^25^. Although our study presented contrasting findings, it is important to note that the inhibitory effects of the MD-1/RP105 complex on TLR4 have been investigated only in B cells and macrophages thus far, with no reported evidence in neutrophils. Divanovic et al. observed that RP105 expression extends beyond B cells in humans, encompassing myeloid cells such as monocytes and neutrophils, where it may exhibit distinct functionality compared to its role in B cells. In the context of myeloid cells, RP105 impedes the assembly of the LPS/TLR4/MD2 complex, thereby exerting inhibitory effects on the immune response triggered by LPS^26^. The aforementioned evidence indirectly suggests that the inhibitory effect of the MD-1/RP105 complex on TLR4 is not constant. Alena Osvaldova et al. reported the highest levels of TLR4 expression in neutrophils, while significant RP105 expression was observed in IgM^+^ B cells, monocytes, and neutrophils, albeit at lower levels than those in B cells. Notably, porcine IgM^+^ B cells exhibited the highest RP105 expression levels, which were at least ten times greater than those in monocytes and neutrophils, suggesting potential distinct roles of RP105 in B cells and myeloid cells^27^. To address this issue, we used an external dataset (GSE3037) and deduced from the analysis of expression profiles (Supplementary Table 11) that the surface expression of TLR4 on neutrophils was significantly greater than that of RP105. Consequently, we postulated that although the MD-1/RP105 complex exerted inhibitory effects on TLR4, its inhibitory potency might not be sufficient to counterbalance the activating influence of MD-1 on TLR4 in carotid-unstable plaques among patients with elevated levels of MD-1 in their peripheral blood.

In HEK293 cells coexpressing TLR4 and MD-1, MD-1 immunoprecipitated with TLR4^28^. The findings of our study demonstrate that MD-1 exhibits direct binding affinity and interaction with TLR4 on neutrophils, subsequently inducing the activation of downstream signaling pathways. We found that incubation of neutrophils with MD-1 results in activation of the MyD88-dependent signaling pathway and that MD-1 activates the Toll-like receptor signaling pathway and induces phosphorylation of MAPKs in a time-dependent manner. In addition, DPI inhibited NETs production but did not affect MAPKs phosphorylation. The mechanism was similarly demonstrated through in vivo experiments, and the crucial role of MD-1 and neutrophils in the formation of unstable plaques in carotid arteries was further elucidated by performing neutrophil depletion experiments.

NETs have been detected in human carotid plaques obtained by CEA^29^. Another analysis showed that NETs were predominantly present in superficial erosions near clusters of apoptotic ECs rather than in lipid-rich vulnerable plaques^30^. NETs can amplify and propagate local processes leading to endothelial injury by activating ECs and increasing adhesion, which in turn promotes additional leukocyte recruitment to the lesion site, thereby enhancing the local inflammatory response^31^. This suggests that the effect of NETs on the stability of carotid plaques may be multifaceted. The microenvironment of unstable plaques is a highly complex physical and biochemical environment involving multiple cells and molecules, such as ECs, inflammatory factors, and chemokines^13^. Pathologic studies have shown that plaque instability is affected by a variety of factors, including inflammation, apoptosis, and angiogenesis^32^. Intraplaque neovascularization has been reported to originate from vascular ECs lining the arterial lumen^33^. No significant differences were detected between HUVECs and HUAECs in terms of the specific characteristics required for successful cardiovascular implant endothelialization, such as proliferation, secretion of vasoactive substances, and extracellular matrix protein production. This suggests that studies using HUVECs likely reflect the behavior of arterial ECs^34^. Kalucka et al., while searching for tissue-specific markers among different vascular beds, identified biomarkers shared by 80–100% of arteries, veins, and lymphatics^35^. Therefore, we believe that both sources of selected ECs (HUVECs and HAECs) can be utilized for in vitro studies. We propose, for the first time, that NETs can induce changes in the plaque microenvironment and subsequently promote neovascularization within the plaque. This process ultimately influences plaque stability. Our proposal is supported by functional analysis of NETs-stimulated HUVECs and HAECs, as well as examination of the raw data from the corresponding dataset (GSE179828). Our findings revealed an intricate response pattern to NETs stimulation in HUVECs, namely, in vitro cell proliferation and diminished migratory capacity, which is directly proportional to the concentration of NETs. In response to stimuli such as inflammation and hypoxia, plaque areas initiate neovascularization as a compensatory mechanism^36^. The process of neovascularization is characterized by incomplete structural development, heightened permeability, fragility, lipid production, and inflammatory cell extravasation, among other factors, ultimately leading to intraplaque hemorrhage^37^. Emerging evidence suggests a positive correlation between intraplaque neovascularization and an elevated risk of plaque rupture. The tube-forming capacity of the two types of ECs (HUVECs and HAECs) has not been systematically summarized in previous studies, and it is likely to vary due to differences in arterial and venous vascular function, reflecting the inherent heterogeneity in endothelial tube formation ability. Therefore, we used both HUVECs and HAECs in vitro for NETs-induced tube formation experiments to mimic neovascularization within plaques stimulated by NETs. Furthermore, our findings were corroborated by clinical plaque specimens and animal experiments, which demonstrated a significant increase in neovascularization within unstable plaques. In our in vitro experiments, we demonstrated that NETs can induce the production of inflammatory factors, including VCAM1, ICAM1, MMP14, VEGFA, and IL6, by HUVECs and HAECs through the NF-κB signaling pathway. These factors are closely associated with intraplaque neovascularization and unstable plaque formation. Abnormal activation of the NF-κB signaling pathway closely correlates with the occurrence and progression of AS, as most genes involved in the inflammatory response and atherosclerotic plaque formation are target genes regulated by NF-κB^38^. Targeting NF-κB and inhibiting the formation of NETs could be promising strategies for improving the microenvironment within plaques.

According to our single-cell sequencing results, MD-1 (*LY86*) exhibited a predominant distribution in macrophages, monocytes, and B cells, consistent with previous findings^11^. Notably, the percentage of M1 macrophages expressing LY86 was significantly greater than that of M2 macrophages, further confirming the crucial role of LY86 in unstable plaque formation. Analysis of differentially expressed genes from the GSE145200 dataset revealed that NETs can stimulate macrophages to produce elevated levels of inflammatory factors such as MD-1 (*LY86*) and VEGFA, thereby establishing a detrimental feedback loop (Supplementary Table 12, Supplementary Fig. 10). This finding highlights the potential for targeting MD-1 to modulate the crosstalk between NETs and unstable plaque formation in carotid arteries. Targeting monocyte-macrophage differentiation may also emerge as a pivotal approach for modulating LY86. LGAL9, SPP1, and AnxA1, which are crucial factors in the interplay between macrophages, monocytes, and B cells, offer novel insights for the future regulation of LY86. The potential role of the transcription factor SPI1 as a prospective regulator of LY86 followed by the targeting of NETs for crosstalk in carotid arteries within unstable plaques warrants further investigation.

In this study, we employed bioinformatics to predict pivotal genes associated with the development of unstable plaques in carotid arteries. Our findings demonstrated that MD-1 (*LY86*) serves as a sensitive inflammatory biomarker that induces neutrophils to generate NETs, which play a crucial role in the plaque microenvironment by stimulating the secretion of various inflammatory factors from ECs and promoting intraplaque neovascularization, thereby destabilizing carotid plaques. Additionally, macrophages initiate the production of additional MD-1 through NETs formation. Ultimately, this positive feedback loop leads to interactions between NETs and ECs and alterations in the plaque microenvironment and ultimately contributes to the formation of unstable plaques in carotid arteries. The main findings of this study are presented in Supplementary Fig. 11; however, it is important to acknowledge the inherent limitations in our research, and the translation of some in vitro findings to in vivo settings may pose challenges; therefore, it is imperative for us to conduct further in vivo investigations to validate and expand upon our findings. Further investigations are warranted to study and target the microenvironment of unstable plaques in the carotid artery, as well as to explore in-depth roles of NETs and the plaque microenvironment in the formation of carotid artery unstable plaques and the involved mechanisms. Additionally, it is crucial to investigate upstream regulatory mechanisms such as the differentiation trajectory and transcription factors of LY86. Among the numerous cytokines utilized for assessing carotid-unstable plaques, MD-1 (*LY86*) may emerge as an inflammatory biomarker that exhibits sensitivity comparable to that of C-reactive protein in future studies.

## Supplementary information

Supplementary material

Supplementary Table 1 Semiquantitative scoring system for histological grading

Supplementary Table 3 deg (different expression genes) GSE41571XGSE28829

Supplementary Table 4 Genes in the purple module

Supplementary Table 5 degXpurple take the genes after intersection

Supplementary Table 6 Neutrophil-related genes

Supplementary Table 9 deg (NETs st. HUVECs) GSE179828

## Appendices

Details are provided in the Supplementary Materials.
