# Author Correction: Neutrophil extracellular traps mediate the crosstalk between plaque microenvironment and unstable carotid plaque formation

**DOI:** 10.1038/s12276-025-01479-0

**Published:** 2025-07-16

**Authors:** Yu Cao, Minghui Chen, Xinyu Jiao, Shuijie Li, Dong Wang, Yongxuan Zhan, Jiaju Li, Zhongfei Hao, Qingbin Li, Yang Liu, Yan Feng, Ruiyan Li, Hongjun Wang, Mingli Liu, Qiang Fu, Yongli Li

**Affiliations:** 1 Department of Neurosurgery, The Second Affiliated Hospital of Harbin Medical University, Harbin, 150086 China; 2 Department of Ultrasound, The Second Affiliated Hospital of Heilongjiang University of Chinese Medicine, Harbin, 150006 China; 3 Department of Biopharmaceutical Sciences, College of Pharmacy, Harbin Medical University, Harbin, 150076 China; 4 State Key Laboratory of Frigid Zone Cardiovascular Diseases (SKLFZCD), Harbin, China; 5 Scientific Research Centre, The Second Affiliated Hospital of Harbin Medical University, Harbin, 150086 China; 6 Department of Chinese Formulae, Heilongjiang University of Chinese Medicine, Harbin, 150040 China

**Keywords:** Computational biology and bioinformatics, Cell biology, Immunology, Molecular biology

Correction to: *Experimental & Molecular Medicine* 10.1038/s12276-024-01281-4, published online 01 August 2024

After the online publication of this article, the authors noticed an error in Figs. 4b and 6a.

Figure assembly errors occurred in Figs. 4b and 6a. The correct figures are presented below:

Figures in the original manuscript:

**Original Figure. 4b** 
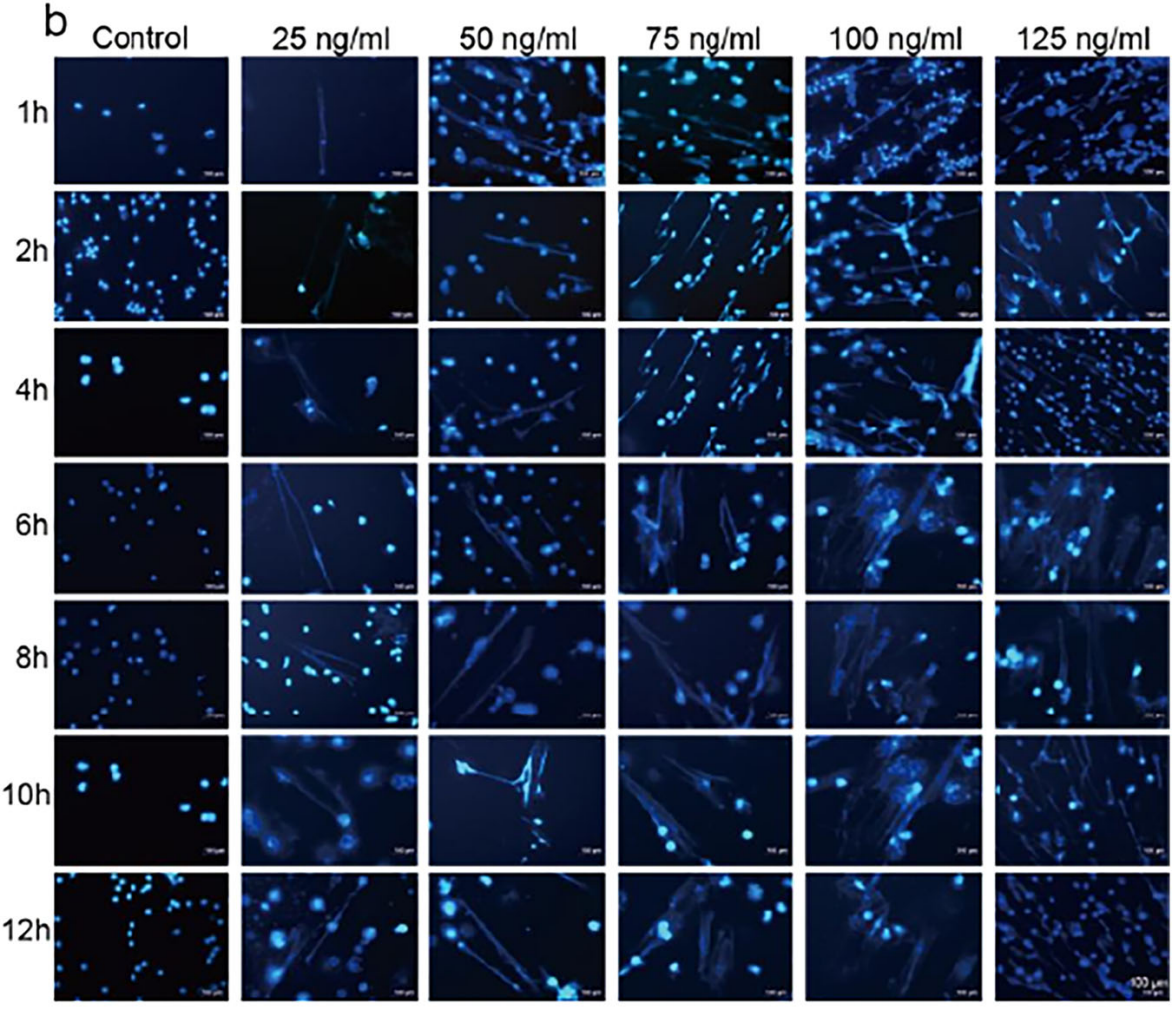


**Original Figure. 6a** 
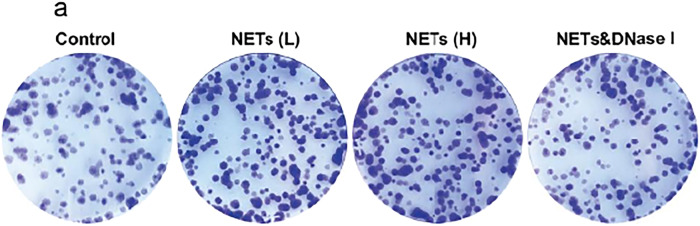


Figures after the changes:

**Revised Figure. 4b** 
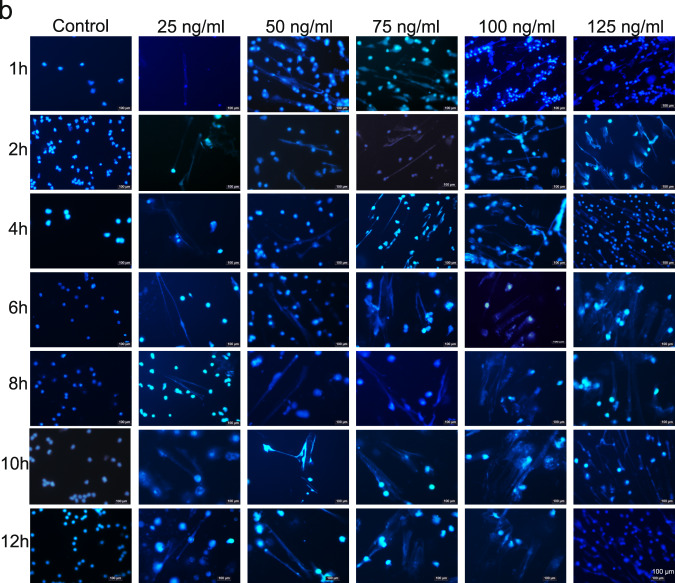


**Revised Figure. 6a** 
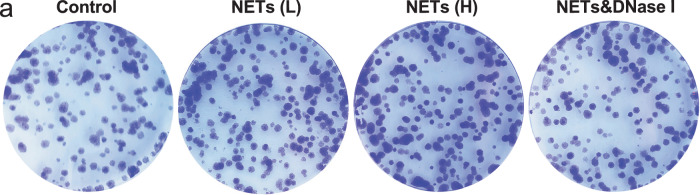


The authors apologize for any inconvenience caused.

The original article has been corrected.

