# Supplementary material for: Neutrophil extracellular traps mediate the crosstalk between plaque microenvironment and unstable carotid plaque formation
Source: Exp Mol Med. 2024 Aug 1;56(8):1717–35. doi: 10.1038/s12276-024-01281-4 (PMC11372095; doi:10.1038/s12276-024-01281-4)
Supplement: Supplementary file 1 — Supplementary material [file 12276_2024_1281_MOESM1_ESM.pdf]

Supplementary material

Gene	Sequence
LY86	F: GTCTCAAGGCTCATCTGTTT
	R: ATGATAGTAGCATTGGCACA
ITGB2	F: CCTCACCCTGTGGCAAGT
	R:TGCTCCAGCGTGTAGGC
CCR1	F: TGGAAACATCCTGGTGG
	R: CAGAAGGGAAGCGTGAA
CSF1R	F: GGTGACCTTGCGATGTG
	R: GCGTTGTTGGTGCTGA
β-actin	F: CACTGTGCCCATCTACGAGG
	R: TAATGTCACGCACGATTTCC

Supplementary Table 2 Primers for quantitative RT-qPCR analysis.

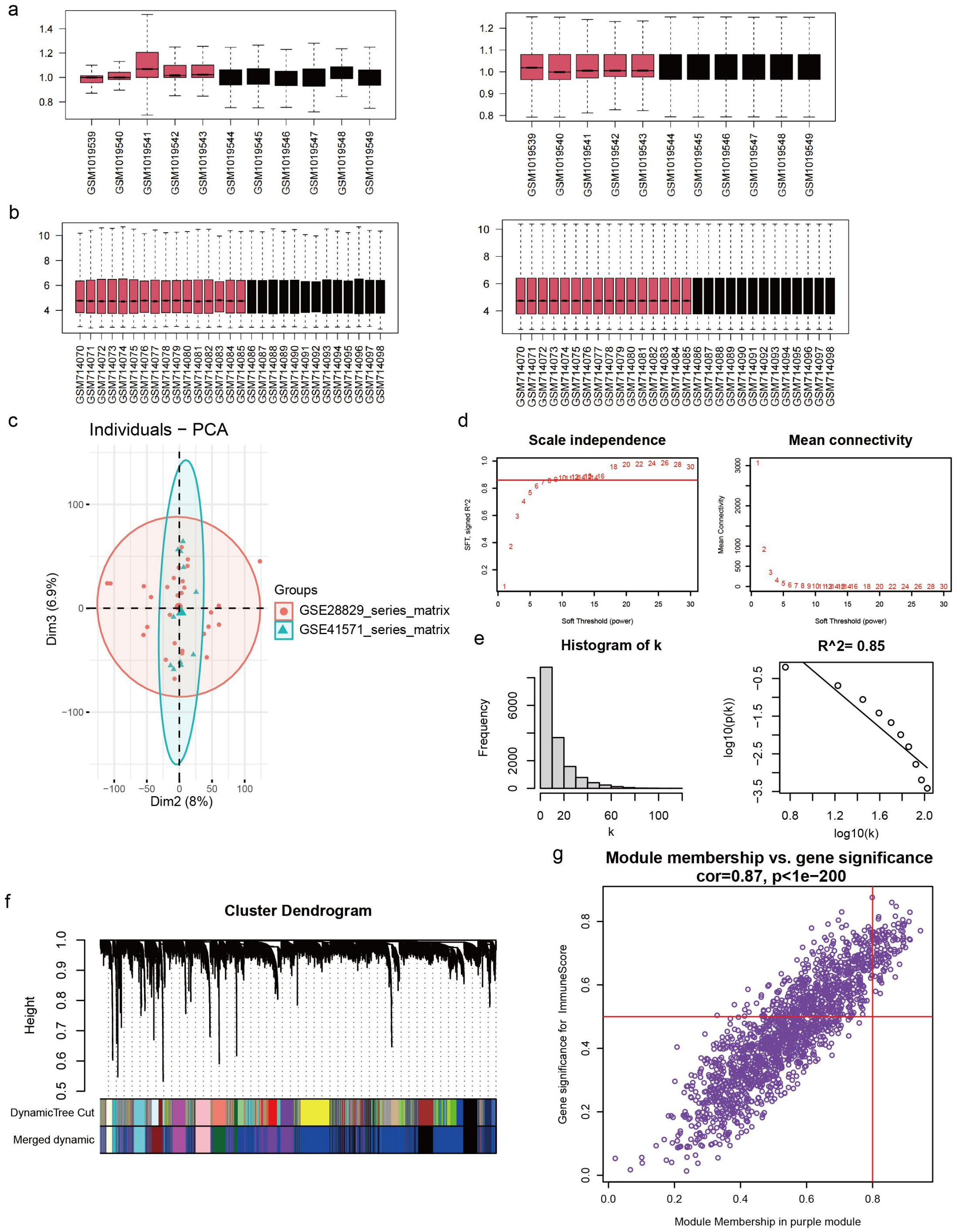

Supplementary Fig. 1 Preprocessing and partial analysis results of the carotid plaque-associated transcriptome dataset using WGCNA. (a) Before and after data correction in the GSE41571 dataset. (b) Before and after data correction in the GSE28829 dataset. The black line in each box represents the median gene expression level, which is almost the same for all samples. Red indicates carotid unstable plaque samples, black indicates carotid stable plaque samples, and the colors are reversed. (c) Principal component analysis of the two datasets before combining the two data sets and removing batch effects. (d) The critical value of soft threshold β is set to 0.85, the red line in the left graph indicates the optimal Powers value of β=8, and the inflection point of the right graph as a reference downtrend is also the optimal Powers value of β=8. (e) Scale-free network. (f) Merged clustering graph. Merged Module is when genes that are closer together (clustered into the same branch) are divided into the same module. (g) Key modules associated with sample traits.

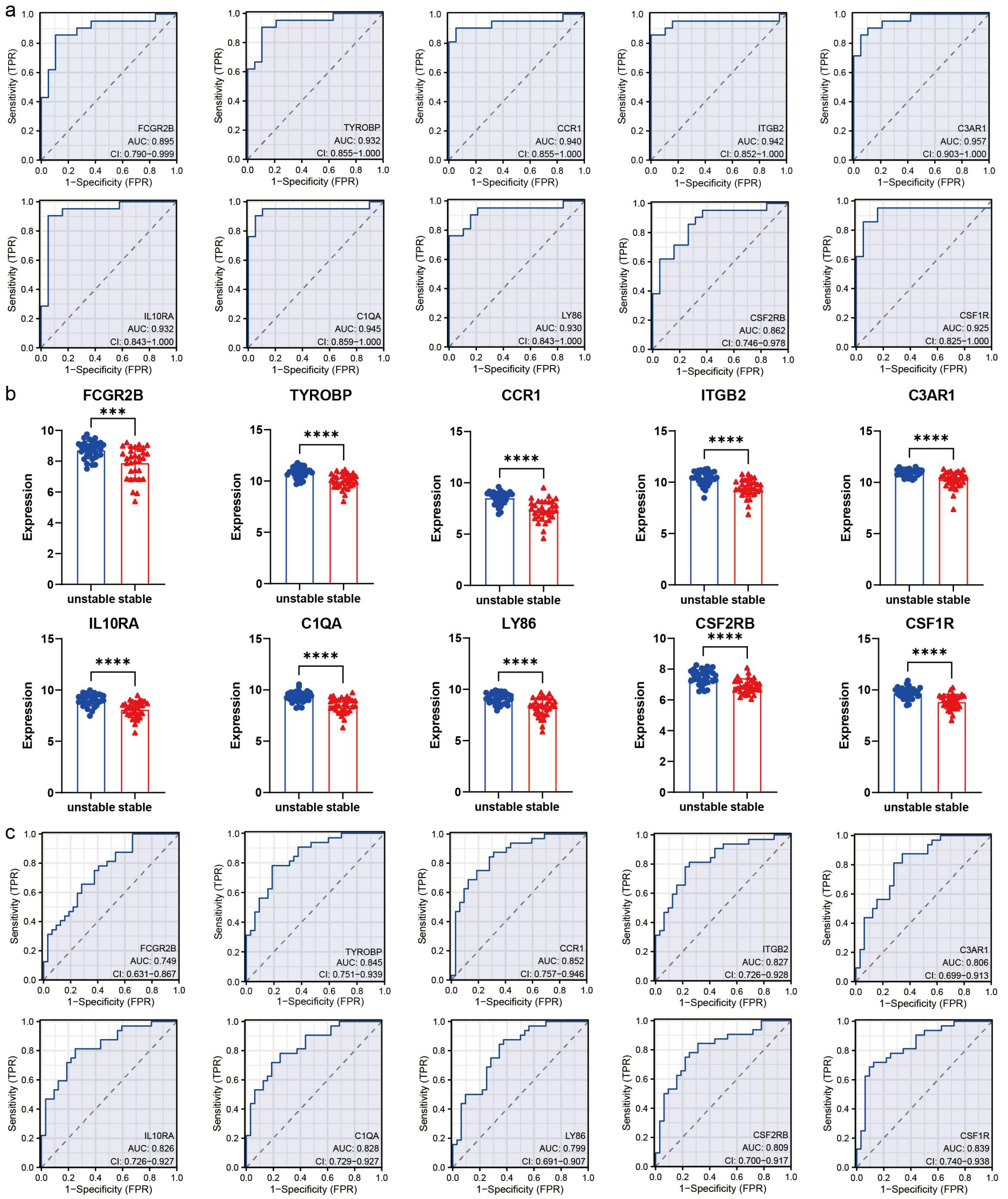

Supplementary Fig. 2 Expression levels and ROC analysis of hub genes. (a) ROC analysis of the hub genes in the experimental dataset. (b-c) Expression levels and ROC analyses of the above genes in the validation dataset.

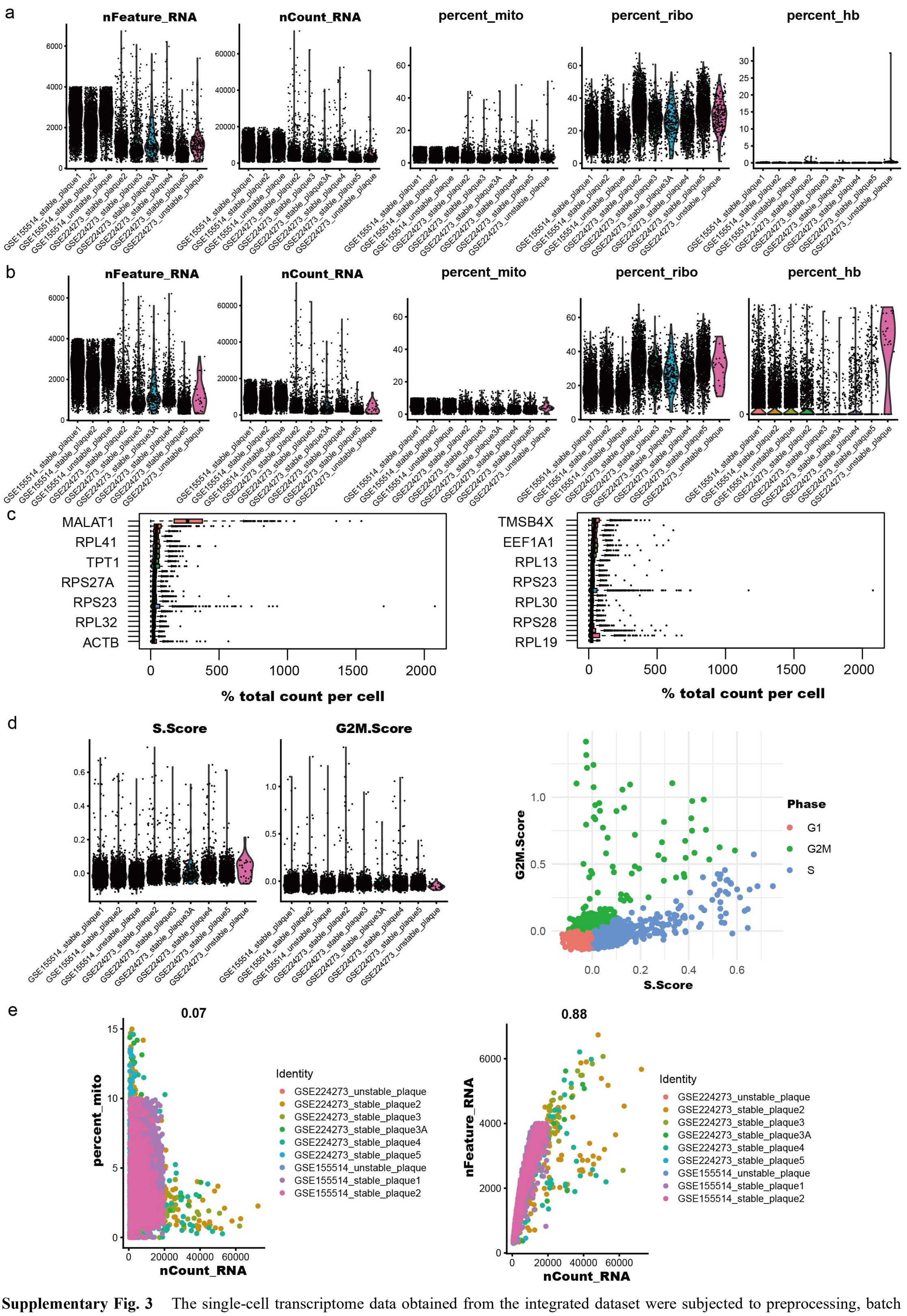

Supplementary Fig. 3 The single-cell transcriptome data obtained from the integrated dataset were subjected to preprocessing, batch effect removal, and filtration. (a) Pre-treatment. (b) Post-treatment. To mitigate the impact of substandard cells on the overall dataset, we applied a stringent filtering process based on nFeaturees RNA, nCount RNA, percent mito, percent ribo, and percent Hb. Evaluation of single cell quality control indicators before and after filtration demonstrates that the information integrity in the preprocessed cells adheres to our predetermined criteria for subsequent analysis. (c) Percentage of each type of gene being filtered in each cell before and after filtration. The count value exceeded 0, and various components such as mitochondria, ribosomes, erythrocytes, and housekeeping

genes were filtered based on the predefined criteria. (d) Filtering and deflation of cell cycle genes. The cell cycle genes were carefully screened and adjusted to minimize their impact on the overall dataset. (e) Correlation of nCount with per cent.MT and nCount with nFeature. The quality of the integrated dataset was ultimately assessed by examining the correlation between nCount and percent.MT, as well as the correlation between nCount and nFeature. Notably, a strong positive correlation of 0.88 was observed between nCount and nFeature, providing compelling evidence for the high quality of the integrated dataset, thereby rendering it suitable for subsequent analyses.

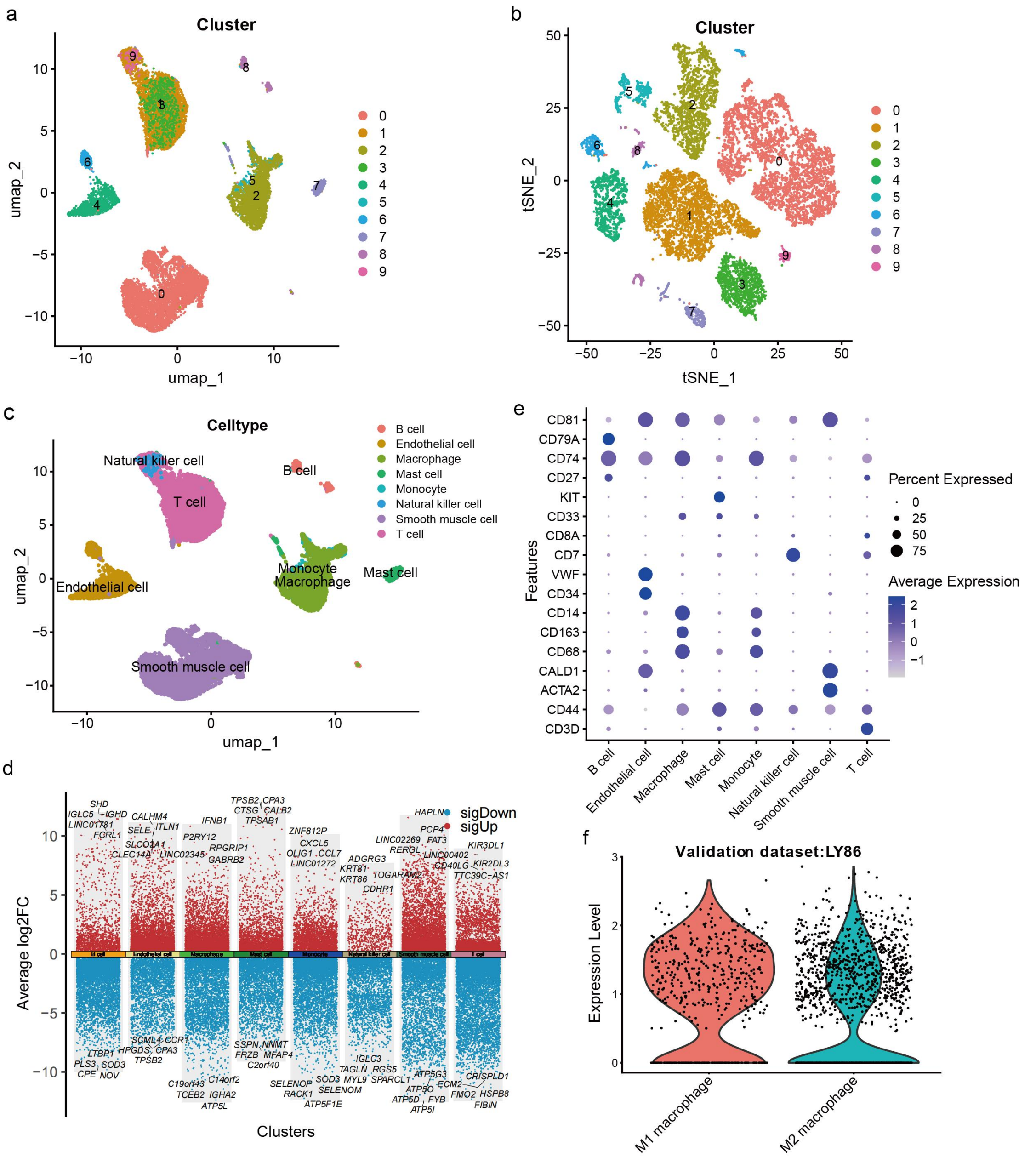

Supplementary Fig. 4 Dimensionality reduction, cell types annotation and validation of integrated single-cell datasets. (a) UMAP dimensionality reduction visualisation diagram. (b) t-SNE dimensionality reduction visualisation map. Dimensionality reduction clustering of integrated data. The t-SNE dimension reduction results revealed a relatively independent distribution of cells within each group, whereas the UMAP analysis demonstrated close proximity between certain clusters, such as cluster 1 and cluster 3, exhibiting noticeable overlap. This suggests the potential need for their combined consideration during further analysis. (c) UMAP display cell clusters annotation results. (d) Volcano plots showed up-regulated and down-regulated TOP5 highly expressed genes in each cell types. (e) Examination of the outcomes derived from cell types annotation. In order to validate the accuracy of cluster annotation, we searched for commonly reported highly specific and highly expressed genes in human cells from various literature sources, including B cells (CD27, CD74, CD79A), endothelial cells (CD34, VWF), macrophages (CD68, CD163, CD14), mast cells (KIT), monocytes (CD68, CD14), natural killer cells (CD7), smooth muscle cells (ACTA2, CALD1), and T cells (CD3D, CD44, CD8A). These genes were used as a reference template to analyze their expression levels in the dataset. The significant increase in expression of each corresponding specific gene indicates that the clustering annotation is more robust. (f) Expression of LY86 in the validation dataset. We replicated the findings using the validation dataset GSE131778 through identical analysis procedures.

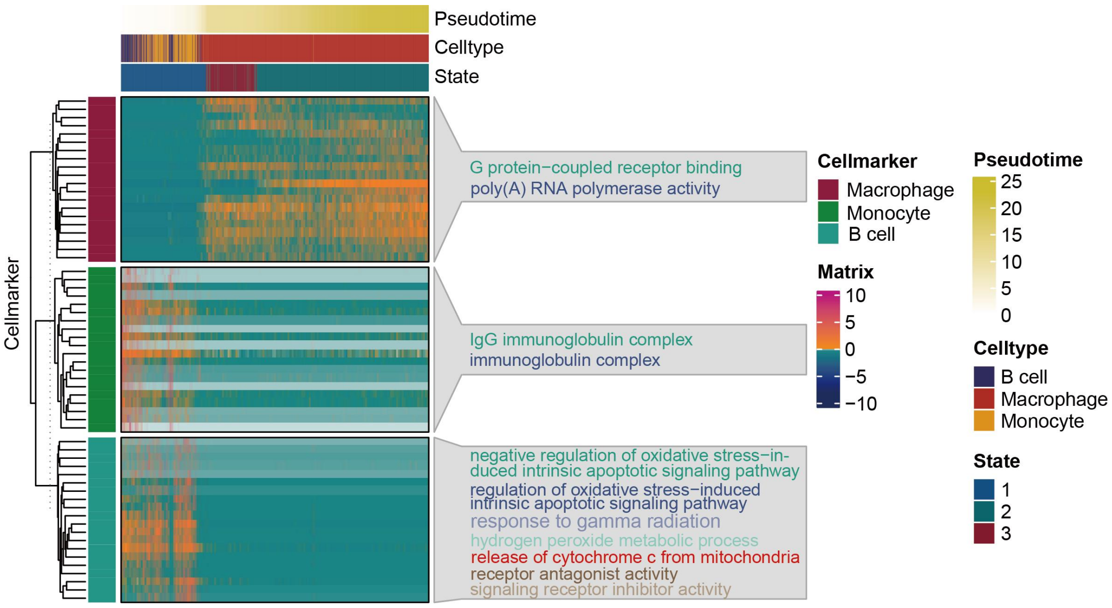

Supplementary Fig. 5 Summary heatmap of LY86 high abundance cell types in the pseudotime analysis based on GO results.

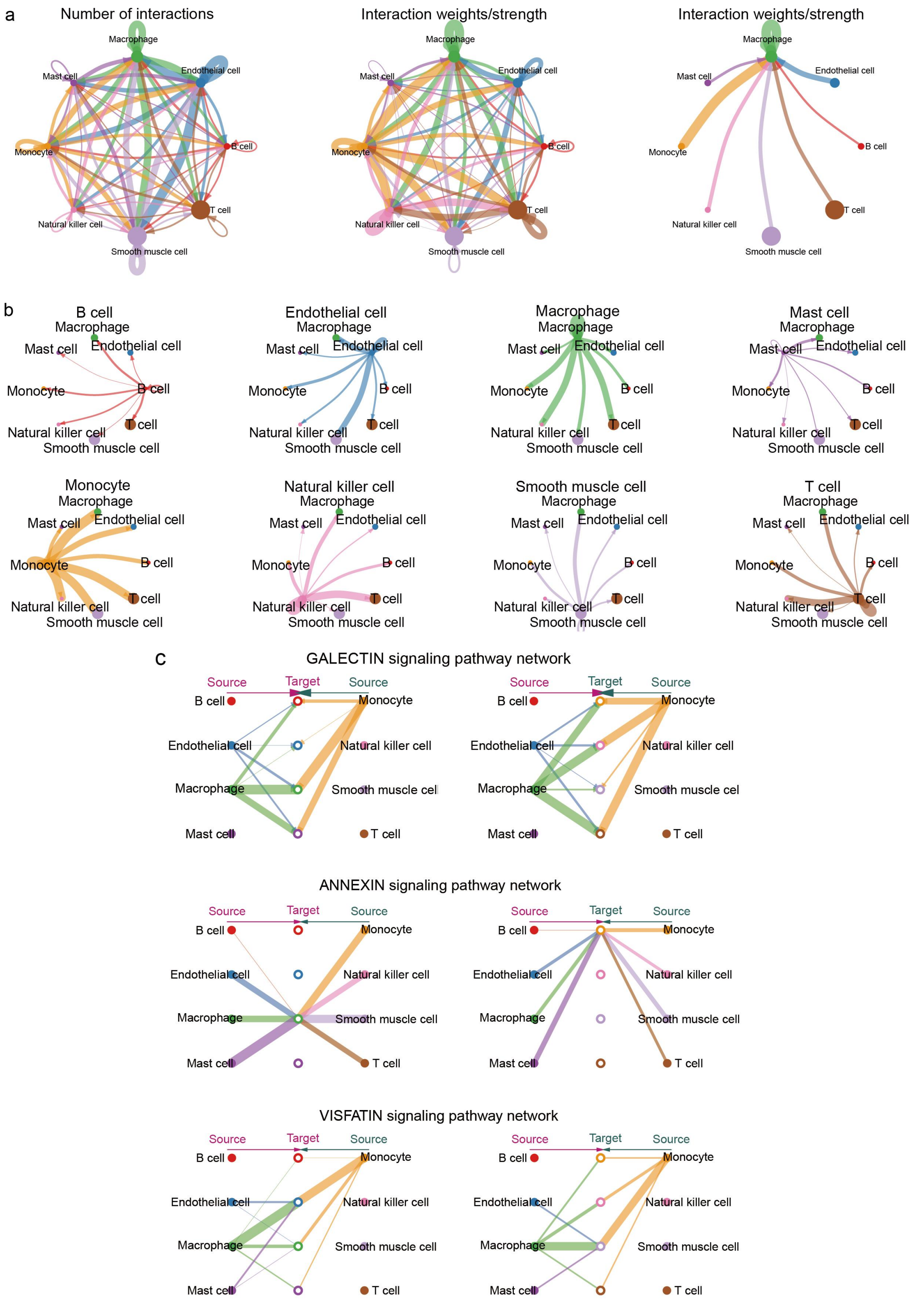

Supplementary Fig. 6 Communication network between all celltypes globally. (a) Shows the number and weight of interactions between the eight major cell types and the weight of interactions between macrophage fine packets and other cells. Each dot represents a cell type whose size is proportional to the number of cells in the type. The thickness of the line connecting the cell types indicates the number of interactions or the net map of weights. The colour of the connecting lines is the same as the colour of the source cell type. The

number on each connecting line indicates the weight of an important ligand-receptor pair between any two pairs of cell types. Loops indicate autocrine. (b) Net plot of the number of interactions between each of the eight cell types with each of the other seven cell types. (c) Common "modes of communication" and signalling pathways between monocytes and macrophages include: the GALECTIN signalling pathway network with LGALS9 as ligand and PTPRC as receptor, the ANNEXIN signalling pathway network with SPP1 as ligand and CD44 as receptor, and the VISFATIN signalling pathway network with ANXA1 as ligand and FPR1 as receptor, etc.

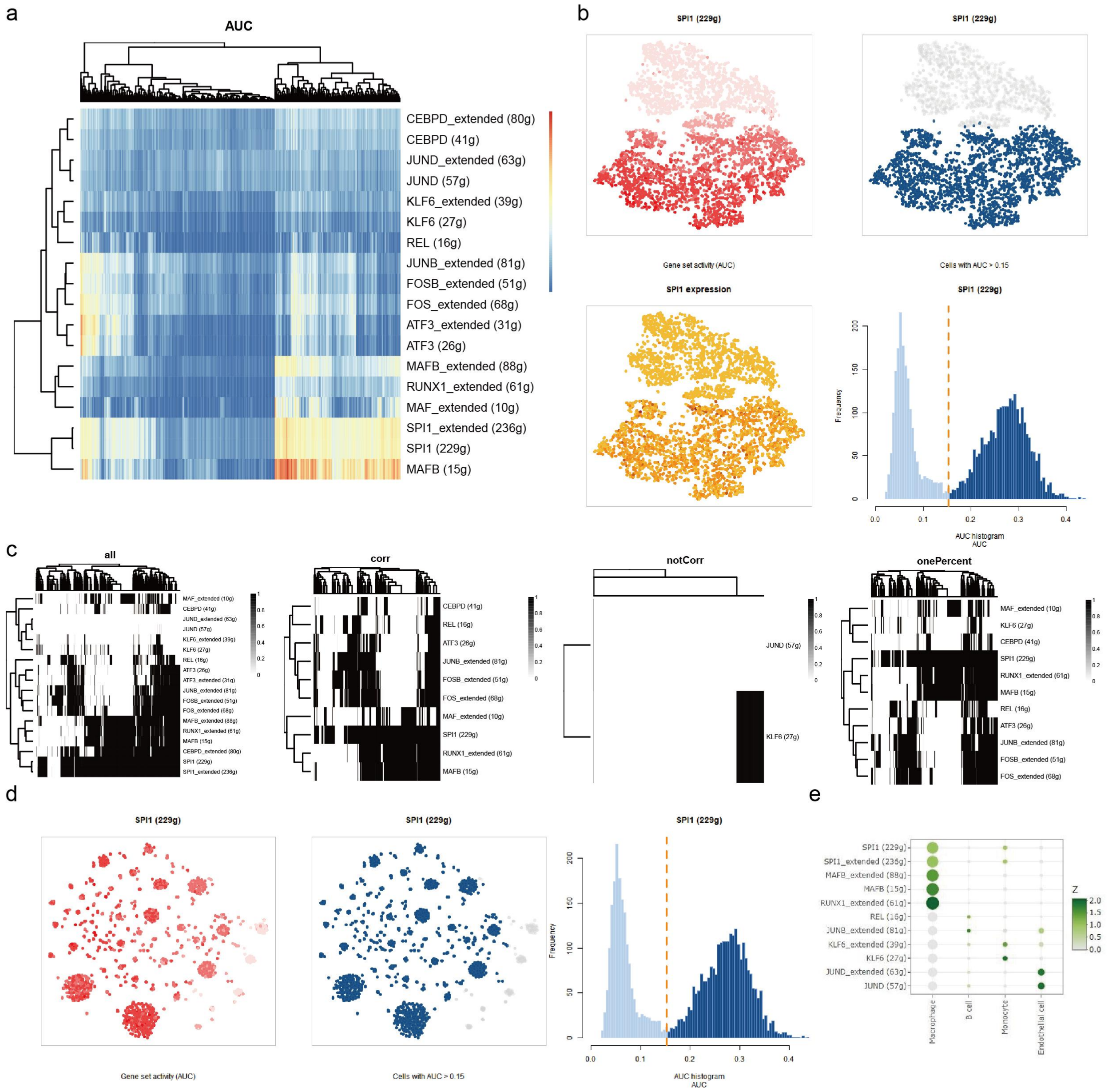

Supplementary Fig. 7 Exploration of the LY86 regulatory network by transcription factor analysis. (a) Heatmap of AUCell score results. Each cell represents the correlation of the transcription factor in the corresponding column and row of the sample, as indicated by a color gradient: blue denotes lower correlation while red signifies higher correlation. The classification tree on the left side describes the cell types with relatively higher expression levels of LY86; a greater number of adjacent rows indicates a higher proportion of LY86 expressed in that particular cell type. (b) t-SNE images of AUC values for each Regulon. The significance of SPI1 is evident in the t-SNE plots, which display t-SNE images depicting regulated levels and color-coded AUC values for each Regulon. Additionally, the t-SNE images illustrating regulated levels and dichotomized AUC values for each Regulon, as well as heatmaps showcasing Regulon expression within t-SNE distributions, further emphasize the importance of SPI1. Furthermore, histograms displaying the distribution of Regulon activity intensity within t-SNE distributions provide additional insights. (c) Heatmap of the activity levels of the dichotomised Regulon modules. The heatmaps, integrating AUCell score with each cell taxon, revealed a strong correlation between the transcription factor SPI1 and LY86. Furthermore, analysis of dichotomised regulon modules demonstrated that SPI1 exhibited the highest relevance to monocytes and macrophages. (d) Regulatory level of dichotomised AUC values for each regulon with coloured t-SNE images. The t-SNE plot, based on the dichotomized Regulon's AUC values, exhibited the highest level of significance. (e) Transcription factor expression specific to each cell type. Consequently, we postulate that the transcription factor SPI1 plays a pivotal role in regulating LY86.

TF	Gene	highConfAnnot	nMotifs	NES	motifDb	spearCor	CoexWeight
CEBPD	LY86	TRUE	4	3.03	10kb	0.46745451	0.047659931
RUNX1	LY86	FALSE	2	4.2	500bp	0.471116465	0.033923579
SPI1	LY86	TRUE	102	12	10kb	0.555417525	0.062430861

Supplementary Table 7 Transcription factors closely related to LY86.

Factor	Unstable Plaques (n=14)	Stable Plaques (n=16)	P value
Age (years)	60.5±7.4292	64.938±6.0494	0.082
Men, n (%)	9 (64.3)	10 (62.5)	1.000
Hypertension, n (%)	12 (85.7)	14 (87.5)	1.000
Current or ex-smoker, n (%)	8 (57.1)	8 (50.0)	0.730
Diabetes mellitus, n (%)	4 (28.6)	4 (25.0)	1.000
Hyperlipemia, n (%)	10 (71.4)	11 (68.9)	1.000
Obesity, n (%)	6 (42.9)	7 (43.8)	1.000
Coronary Artery Disease, n (%)	12 (85.7)	13 (81.3)	1.000
Family history of stroke, n (%)	4 (28.6)	4 (25.0)	1.000
Kidney Disease, n (%)	3 (21.4)	4 (25.0)	1.000
Side of Plaque (left), n (%)	10 (71.4)	11 (68.8)	1.000
Side of Plaque (Bilateral), n (%)	4 (28.6)	3 (18.8)	0.675
Frequent Neurological Symptoms, n (%)	14 (100.0)	14 (87.5)	0.485

Supplementary Table 8 Basic clinical characteristics of the 30 enrolled patients.

Gene Symbol	Function
VCAM1<br>(Vascular cell adhesion protein 1)	Cell adhesion glycoproteins are mainly expressed on the surface of endothelial cells and play an important role in immune surveillance and inflammatory responses[1]. By interacting with different types of integrins, they act as major regulators of leukocyte adhesion to the endothelium[2]. In the inflammatory response, binding to ligands on the surface of activated endothelial cells activates calcium channels and the plasma membrane-associated small GTPase RAC1, leading to leukocyte migration across the endothelium[3]. It can also serve as a quality control checkpoint for entry into the bone marrow by providing a "don't eat me" imprint in the context of major histocompatibility complex (MHC) class i expression[4].
ICAM1<br>(Intercellular adhesion molecule 1)	ICAM proteins are ligands for the leukocyte adhesion protein LFA-1 (integrin α-l/β-2). During leukocyte migration across the endothelium, ICAM1 is involved in facilitating the assembly of endothelial cell parietal cups through activation of ARHGEF26/SGEF and RHOG[5].
MMP14<br>(Matrix metalloproteinase-14)	Degradation of various components of the extracellular matrix such as collagen. Activation of procollagenase a. is essential for pericellular collagenolysis and modeling of skeletal and extraskeletal connective tissues during development[6].

VEGFA (Vascular endothelial growth factor A)	Growth factor activity in angiogenesis, vasculogenesis and endothelial cell growth. Induces endothelial cell proliferation, promotes cell migration, inhibits apoptosis, and induces vascular permeability. Binds to FLT1/VEGFR1 and KDR/VEGFR2 receptors, heparan sulfate and heparin[7].
IL6 (Interleukin-6)	Acts as an important factor in bone homeostasis by inducing VEGF to act directly or indirectly on the vasculature, leading to increased angiogenic activity and vascular permeability[8]. Induces inflammation-induced epithelial regeneration through activation of the IL6ST-YAP-NOTCH pathway[9].

Supplementary Table 10 Functional profiles of hub genes in HUVECs and HAECs.

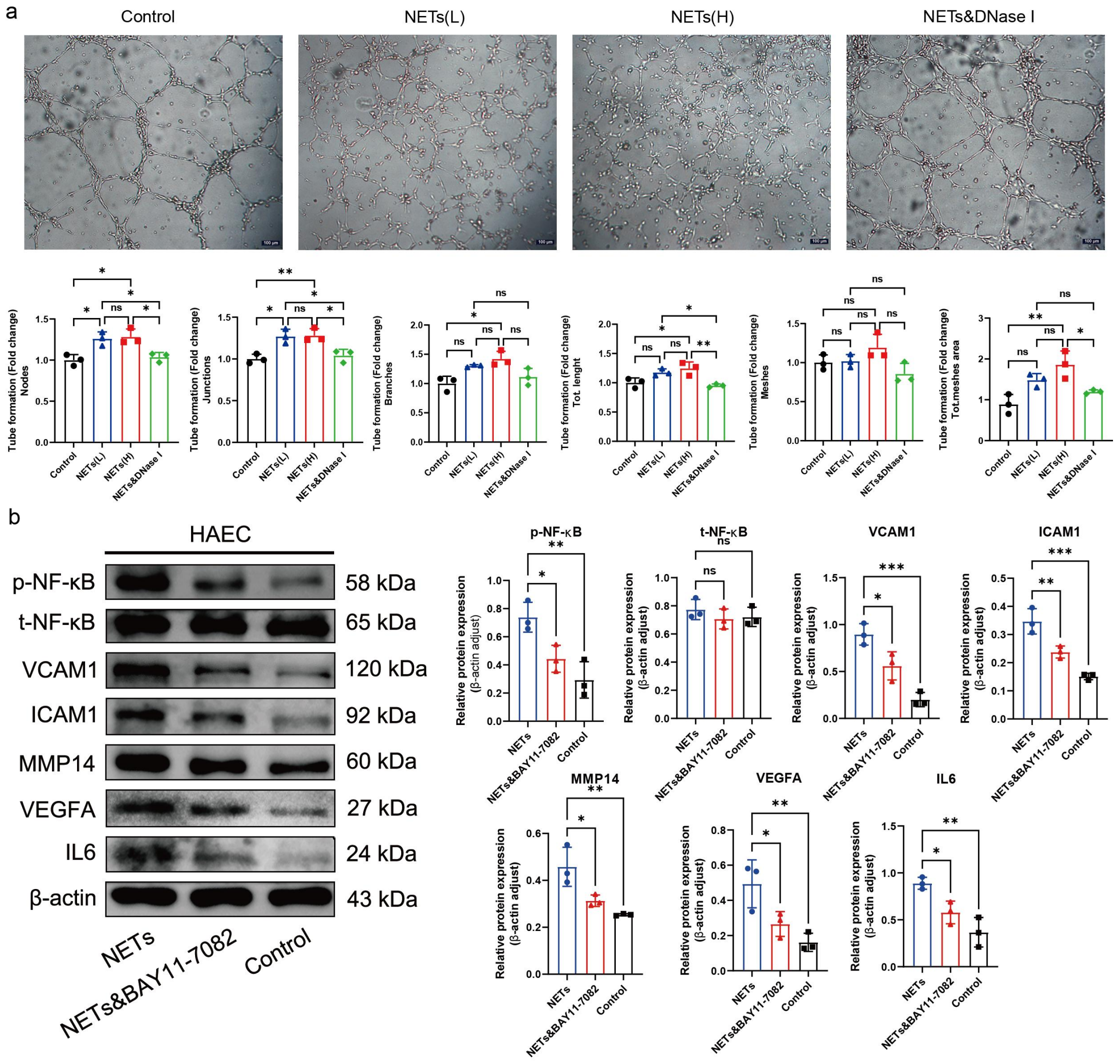

Supplementary Fig. 8 Results from the HAEC-related tube-forming assay and Western blot analysis were obtained. (a) Tube forming assays treated with HAEC in the above manner (Scale bar = 100 μm). (b) Western blot showing the expression of VCAM1, ICAM1, MMP14, p-NF-κB, t-NF-κB, VEGFA and IL6 in HAEC under different conditions.

t: total value; p: phosphorylation value. β-actin was used as an internal control.

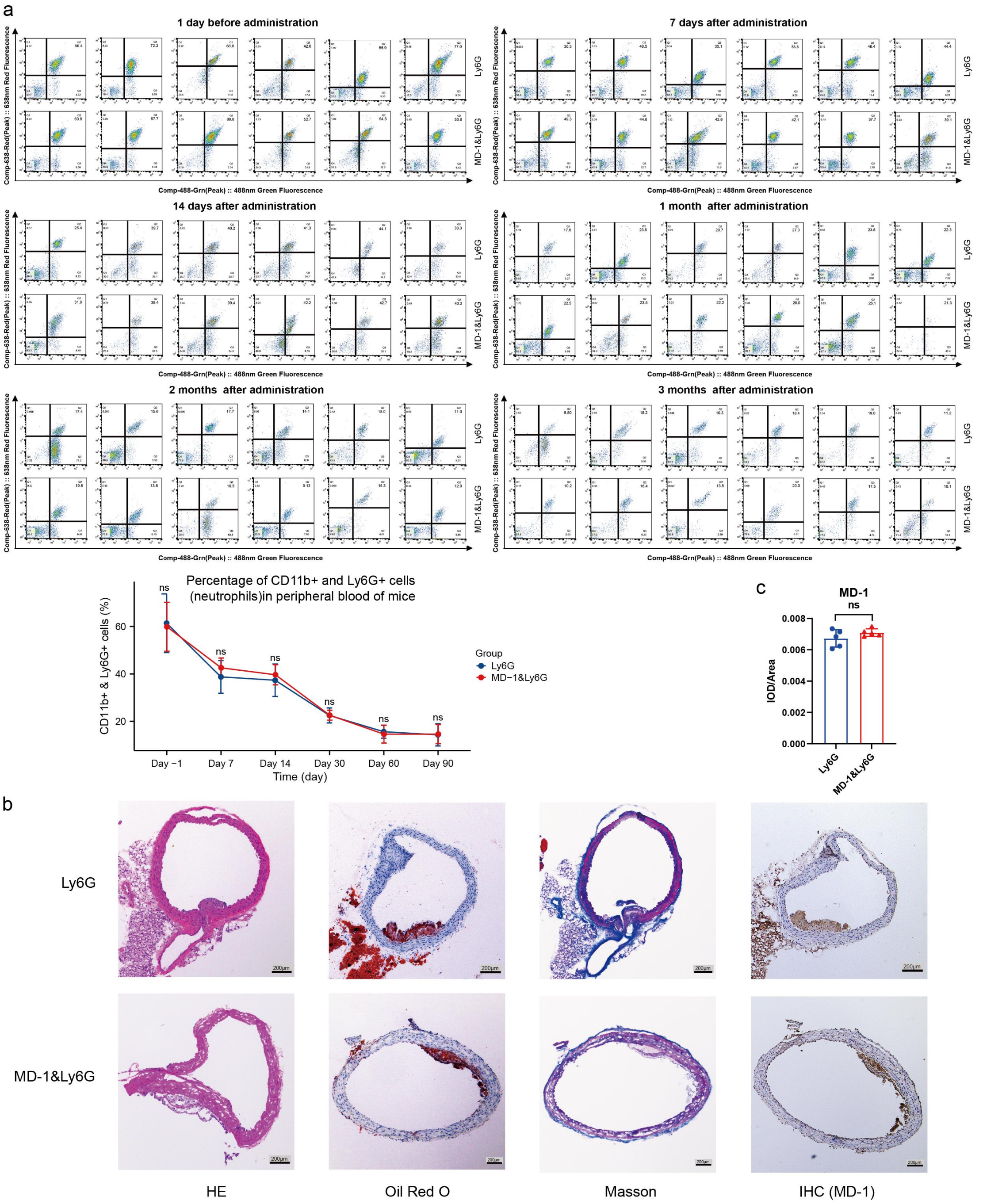

Supplementary Fig. 9 Relevant validation of animal models of neutrophil depletion. (a) Detection of peripheral blood neutrophil depletion in mouse neutrophil depletion model group at different time points by flow cytometry. (b) Pathological staining results of mouse neutrophil depletion model group. (c) Quantification of MD-1 within the plaques of mice in the neutrophil depletion model group was performed by immunohistochemical staining and representative images of both groups are shown.(Scale bar = 100 μm)

Sample	TLR4	RP105
GSM66867	672.52195	4.598958333
GSM66868	114.2070627	5.094166667
GSM66869	1021.908992	4.821666667
GSM66870	1013.625776	4.442291667
GSM66871	570.2308632	4.65625
GSM66872	178.5890659	5.291666667
GSM66873	346.2545876	6.4575
GSM66874	21.11317814	4.904166667
GSM66875	364.7768162	5.286666667
GSM66876	141.5173221	5.03625
GSM66877	1279.404203	4.407916667
GSM66878	817.9672588	4.88125
GSM66879	543.0051318	5.593541667
GSM66880	421.1606871	4.925416667
GSM66881	519.7889459	5.660833333
GSM66882	22.1417771	4.987083333
GSM66883	468.4193027	4.402083333
GSM66884	148.98065	5.034166667
GSM66885	1005.734458	4.366666667
GSM66886	1030.60184	5.067291667
GSM66887	776.6394894	5.0975
GSM66888	322.7813649	4.715
GSM66889	946.8155203	5.033885598
GSM66890	15.71479925	4.960754286

Supplementary Table 11 Expression of TLR4 and RP105 per sample in the GSE3037 dataset.

Symbol	BL2_NETs_pos	BL4_NETs_pos	BL1_NETs_pos	BL1_NETs_neg	BL1_NETs_neg	BL1_NETs_neg
MD-1 (*LY86*)	3776	5031	3614	2771	4482	1793
VEGFA	1686	266	543	296	314	439

Supplementary Table 12 Expression of MD-1 (*LY86*) and VEGFA in the GSE145200 dataset.

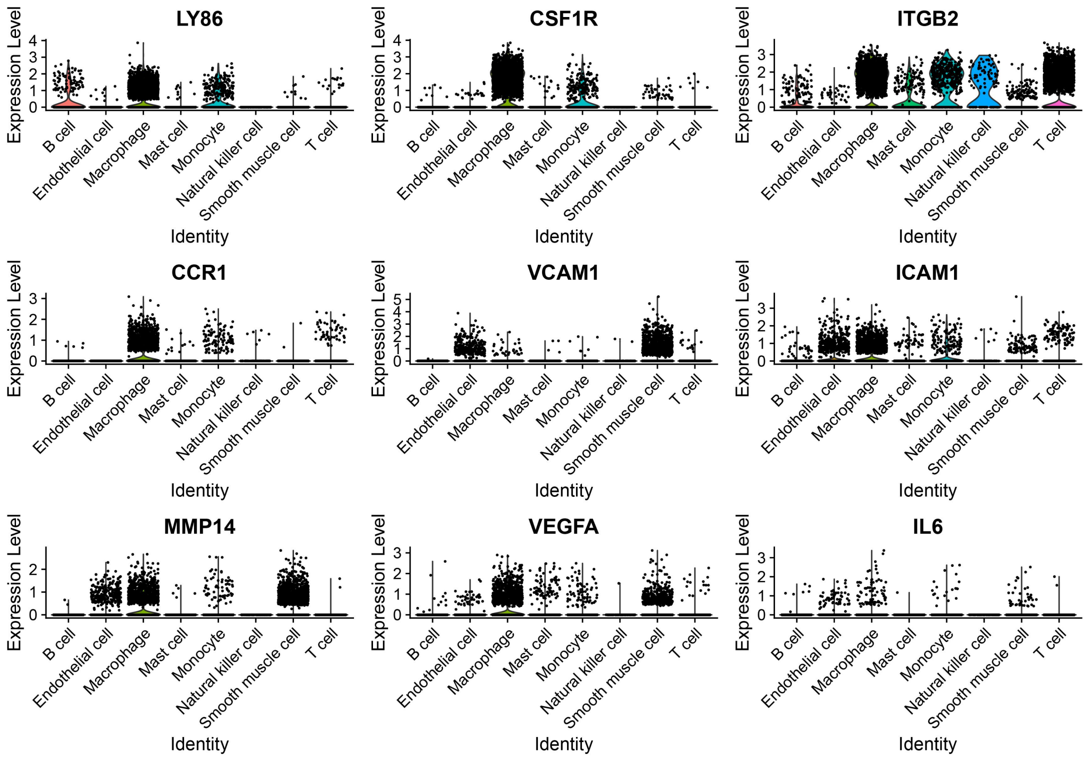

Supplementary Fig. 10 Expression of genes associated with the formation of carotid unstable plaques in various cell types based on single-cell transcriptomic data.

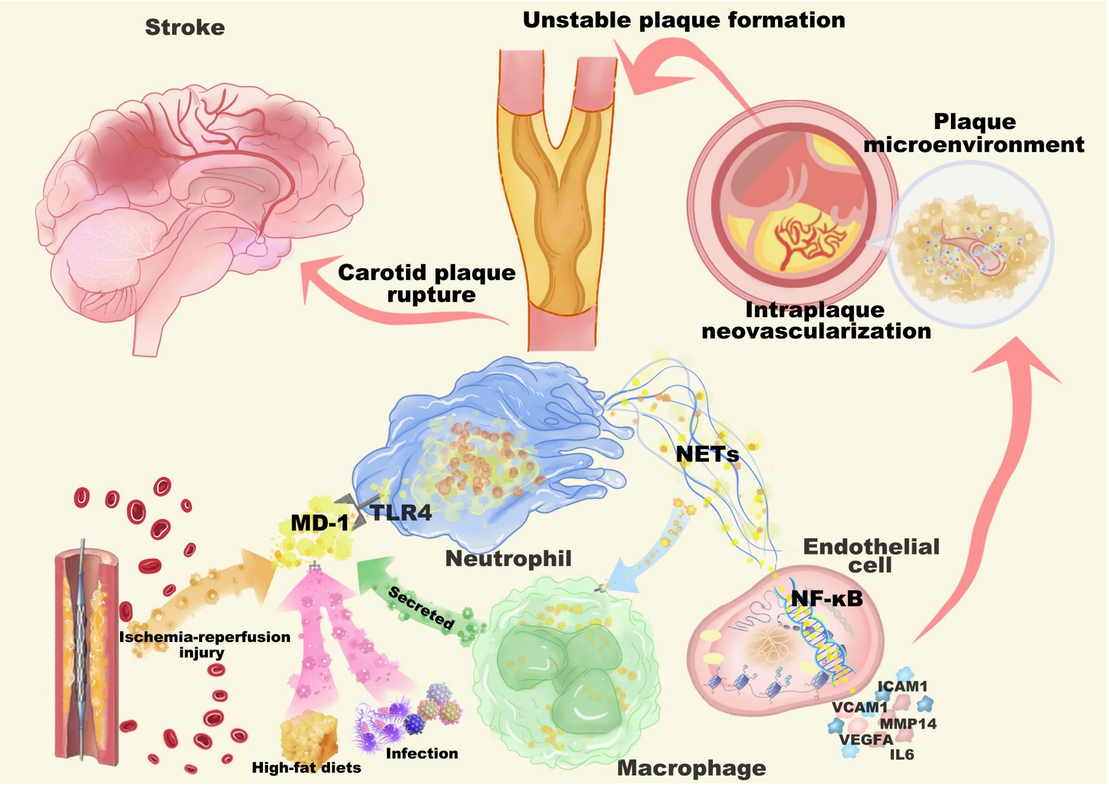

Supplementary Fig. 11 Schematic representation illustrating the primary research mechanisms pertaining to this topic.

Supplementary references

1. Li, Y. et al. TRIM65 E3 ligase targets VCAM-1 degradation to limit LPS-induced lung inflammation. *J Mol Cell Biol*. **12**(3), 190–201 (2020).

2. Taooka, Y., Chen, J., Yednock, T., & Sheppard, D. The integrin alpha9beta1 mediates adhesion to activated endothelial cells and transendothelial neutrophil migration through interaction with vascular cell adhesion molecule-1. *J Cell Biol*. **145**(2), 413–420 (1999).

3. Marchese, M. E., Berdnikovs, S., & Cook-Mills, J. M. Distinct sites within the vascular cell adhesion molecule-1 (VCAM-1) cytoplasmic domain regulate VCAM-1 activation of calcium fluxes versus Rac1 during leukocyte transendothelial migration. *Biochemistry*. **51**(41), 8235–8246 (1999).

4. Pinho, S. et al. VCAM1 confers innate immune tolerance on haematopoietic and leukaemic stem cells. *Nat Cell Biol*. **24**(3), 290–298 (2022).

5. van Buul, J. D. et al. RhoG regulates endothelial apical cup assembly downstream from ICAM1 engagement and is involved in leukocyte trans-endothelial migration. *J Cell Biol*. **178**(7), 1279–1293 (2022).

6. Golubkov, V. S. et al. The Wnt/planar cell polarity protein-tyrosine kinase-7 (PTK7) is a highly efficient proteolytic target of membrane type-1 matrix metalloproteinase: implications in cancer and embryogenesis. *J Biol Chem*. **285**(46), 35740–35749 (2010).

7. Glorioso, N. et al. Association of ATP1A1 and dear single-nucleotide polymorphism haplotypes with essential hypertension: sex-specific and haplotype-specific effects. *Circ Res*. **100**(10), 1522–1529 (2007).

8. De Benedetti F. The impact of chronic inflammation on the growing skeleton: lessons from interleukin-6 transgenic mice. *Horm Res*. **72** Suppl 1, 26–29 (2009).

9. Nakahara, H. et. Al. Anti-interleukin-6 receptor antibody therapy reduces vascular endothelial growth factor production in rheumatoid arthritis. *Arthritis Rheum*. **48**(6), 1521–1529 (2003).
